# Phosphorylation-dependent BRD4 dimerization and implications for therapeutic inhibition of BET family proteins

**DOI:** 10.1038/s42003-021-02750-6

**Published:** 2021-11-09

**Authors:** Francesca Malvezzi, Christopher J. Stubbs, Thomas A. Jowitt, Ian L. Dale, Xieyang Guo, Jon P. DeGnore, Gianluca Degliesposti, J. Mark Skehel, Andrew J. Bannister, Mark S. McAlister

**Affiliations:** 1 Structure, Biophysics and Fragment-Based Lead Generation, Discovery Sciences, BioPharmaceuticals R&D, AstraZeneca, Cambridge, UK; 2 Wellcome Trust Centre for Cell-Matrix Research, University of Manchester, Manchester, UK; 3 Discovery Biology, Discovery Sciences, BioPharmaceuticals R&D, AstraZeneca, Cambridge, UK; 4 Structure, Biophysics and Fragment-Based Lead Generation, Discovery Sciences, BioPharmaceuticals R&D, AstraZeneca, Gothenburg, Sweden; 5 Mechanistic Biology & Profiling, Discovery Sciences, BioPharmaceuticals R&D, AstraZeneca, Boston, USA; 6 Biological Mass Spectrometry and Proteomics, MRC Laboratory of Molecular Biology, Francis Crick Avenue, Cambridge, UK; 7 The Gurdon Institute and Department of Pathology, University of Cambridge, Cambridge, UK; 8 Present Address: Molecular Partners AG, Schlieren, Switzerland

**Keywords:** Biophysics, Structural biology, Supramolecular assembly

## Abstract

Bromodomain-containing protein 4 (BRD4) is an epigenetic reader and oncology drug target that regulates gene transcription through binding to acetylated chromatin via bromodomains. Phosphorylation by casein kinase II (CK2) regulates BRD4 function, is necessary for active transcription and is involved in resistance to BRD4 drug inhibition in triple-negative breast cancer. Here, we provide the first biophysical analysis of BRD4 phospho-regulation. Using integrative structural biology, we show that phosphorylation by CK2 modulates the dimerization of human BRD4. We identify two conserved regions, a coiled-coil motif and the Basic-residue enriched Interaction Domain (BID), essential for the BRD4 structural rearrangement, which we term the phosphorylation-dependent dimerization domain (PDD). Finally, we demonstrate that bivalent inhibitors induce a conformational change within BRD4 dimers in vitro and in cancer cells. Our results enable the proposal of a model for BRD4 activation critical for the characterization of its protein-protein interaction network and for the development of more specific therapeutics.

## Abstract (web summary)

Malvezzi et al. discuss the impact of BRD4 phosphorylation on the formation of dimers and identify the key residues necessary for this dimerization. They also discuss the differential role of monovalent and bivalents bromodomain inhibitors regarding the interaction with these dimers and suggest a new model of BRD4 binding to chromatin.

## Introduction

BRD4 (BRomoDomain protein 4) is an epigenetic reader belonging to the BET (Bromodomain and Extra-Terminal domain) protein family, which also includes BRD2, BRD3, and BRDT (BRomoDomain protein Testes-specific)^1^. BRD4 has key functions in multiple processes including transcriptional regulation^1^, DNA damage response^2^, and virus maintenance and replication^3^. At the basis of most BRD4 functions is the ability to bind to acetylated chromatin through bromodomain 1 (BD1) and bromodomain 2 (BD2) that are located in tandem within the N-terminal part of the protein. These are highly conserved 110 amino acid (aa) domains composed of a bundle of four helices separated by two loops that form a hydrophobic pocket for interaction with mono- or di-acetylated peptides^4^. Despite the high sequence identity between the two bromodomains, they recognize different epigenetic marks: BD1 binds in vitro to mono- and multiply-acetylated H4 peptides, while BD2 exhibits promiscuous interaction with both acetylated H3 and H4 histone tails^4^ and it can also bind to acetylated transcription factors, such as TWIST1^5^. All members of the BET family also contain an extra-terminal domain (ET), which in BRD4 has been demonstrated to interact with multiple binding partners and influence gene transcription^6^. Finally, the long isoforms of BRD4 and BRDT display an intrinsically disordered region, shown to form in BRD4 phase-separated droplets at the chromatin to compartmentalize transcription^7^, and a conserved C-Terminal Motif that, together with BD2, contributes to activate transcription of targeted genes by recruiting the positive transcriptional elongation factor b^8,9^. It has been recently reported that the BRD4 isoform C (aa 1-722), which lacks the long C-terminal intrinsically disordered region and has the last three residues ETA substituted by GPA, is also able to form liquid-like condensates at the nucleus, similarly to the long isoform A (aa 1–1362). The two isoforms seem to modulate the expression of a subset of genes in an opposite fashion^10,11^ and have been demonstrated to locate to distinct nuclear compartments^12^.

The discovery that the transcription of *c-MYC* and other oncogenic genes is regulated by BRD4^13^ and that selective inhibition of BET bromodomains with small molecules, JQ1 and I-BET, is effective against various hematological cancers^14–18^, encouraged further development of BET inhibitors towards the clinic. BET inhibitors act by binding to the acetylated lysine binding pockets of BD1 and BD2 and disrupting interactions with chromatin and transcription factors, thus suppressing transcription of *c-MYC* and other proto-oncogenes. Although the majority of BET inhibitors bind to both BD1 and BD2, specific compounds targeting either BD1 or BD2 of BET proteins were recently developed^19,20^. Importantly, the efficacy of some of the BET inhibitors against hematological and solid tumors has been demonstrated in pre-clinical studies^21^, and they are also of interest in inflammatory and viral diseases. Although the inhibition of BRD4 is likely to be the main target of BET inhibitors, it has to be stressed that these small-molecules bind to all members of the BET protein family and that specific inhibitor of each member has been difficult to identify. Recently, several bivalent BET inhibitors (biBETs) were developed by three distinct groups, which are able to target two bromodomains (BD1 or BD2) simultaneously and show higher potencies and efficacies compared to monovalent counterparts^22–25^.

Despite the broad therapeutic interest, the molecular details of BRD4 function and regulation are not fully understood. Phosphorylation of BRD4 by casein kinase 2 (CK2) is necessary for active gene transcription and controls the activity of BRD4 by positively regulating its binding to acetylated chromatin, as well as to human p53 and viral E2 transcription factors^26,27^. In addition, hyperphosphorylation of BRD4 has been identified as a resistance mechanism in triple-negative breast cancer against BET inhibition due to an increased p-BRD4-mediated recruitment of the Mediator complex, a multi-protein activator of RNA pol II^28^. As in many targets of CK2, BRD4 harbors multiple highly conserved consensus sites for CK2 phosphorylation (S/TxxE/D, where x is any residue) that are located in two main clusters: one, named N-terminal phosphorylation sites (NPS) downstream of BD2, and another, C-terminal phosphorylation sites (CPS), after the ET domain. A proposed phospho-regulation mechanism involves a conformational switch driven by the NPS^29^. It was suggested that the unphosphorylated NPS interacts with BD2 to inhibit chromatin binding. Upon phosphorylation by CK2, NPS was proposed to bind to a lysine-rich region immediately downstream, called BID (Basic-residue enriched Interaction Domain), thereby releasing auto-inhibition of BD2 and allowing chromatin interaction. Although the “phospho-switch” is an elegant and simple model for the regulation of BRD4 activity, there are currently no structural or biophysical reports in its support.

Here, we provide insights into the phospho-regulation of human BRD4. Using an integrative structural biology approach, we demonstrate that BRD4 dimerizes upon phosphorylation of NPS by CK2. We identify BID and a conserved coiled-coil region downstream of the bromodomains as required for dimerization. Finally, we show the effects of biBETs on the BRD4 conformation in vitro and in cellular NanoBRET assays. Guided by our analyses, we propose a revised model for the regulation of BRD4 in which phosphorylation modulates the conformation and oligomeric state of the protein, thus creating a multi-valent platform for co-localization of transcriptional complexes. This work not only provides a key for the interpretation of phospho-regulated protein-protein interactions of BRD4, but it also gives mechanistic insight into the control of BRD4 activity while underlining the importance of biophysical and structural data on physiologically relevant constructs in the understanding of protein functional mechanisms.

## Results

### Dimerization of BRD4 is driven by phosphorylation and requires the BID region

**Fig. 1:**
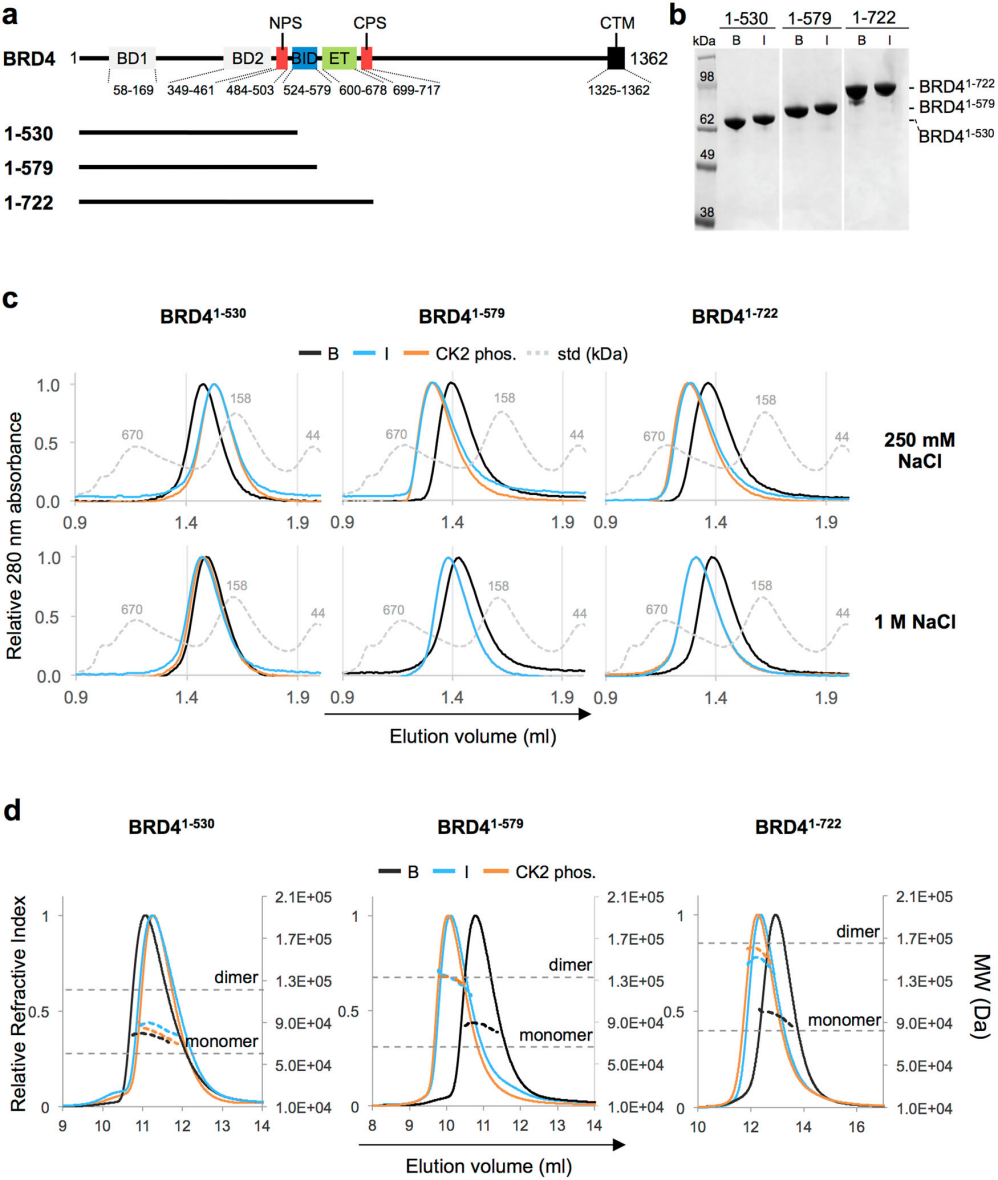
Dimerization of BRD4 constructs containing BID upon CK2 phosphorylation. **a** Summary of the BRD4 constructs used in the study. A schematic representation of full-length BRD4 (long isoform A) and known regions is reported. **b** Coomassie-stained SDS-PAGE gel showing the purity of the recombinantly produced BRD4 constructs 1-530, 1-579, 1-722. **c** Elution profiles of analytical size-exclusion chromatography (SEC) performed with 20 µM of the indicated constructs in the presence of 250 mM NaCl or 1 M NaCl. Std: Gel filtration standards (Bio-Rad) analysed in the corresponding low or high salt running buffer. **d** Elution profiles of the indicated constructs analysed by SEC-MALS. Samples are labeled as follows: black line indicates purified from bacteria, blue line indicates purified from insect cells, orange line indicates phosphorylated in vitro using CK2, dotted grey line in panels **c**, and **d** indicate protein molecular weight standards. The dotted line at each peak, colored as above, indicates the measured MW. The dotted horizontal grey lines in panel d represent the theoretical MW of the monomer or dimer of BRD4^1–530^, BRD4^1–579^, BRD4^1–722^, calculated from the primary sequence.

To dissect the effects of phosphorylation on the structure of BRD4, we used three previously described constructs^29^: 1) BRD4^1–530^, encompassing BD1, BD2, and NPS; 2) BRD4^1–579^, which further includes BID; 3) BRD4^1–722^, which additionally spans the ET domain and the CPS region, and comprises the isoform C (Fig. 1a). Attempts to obtain protein samples of isoform A (aa 1–1362), failed due to low expression levels in insect cells and proteolytic instability.

Unphosphorylated proteins were produced by expression in *E. coli*, while phosphorylated samples were generated by expression in insect cells or by in vitro CK2-mediated phosphorylation. Purified proteins were obtained from both bacteria and insect cells (Fig. 1b) and multiply phosphorylated sites were observed by mass spectrometry from insect cells and in vitro phosphorylated samples (Supplementary Fig. 1 and 2). A single acetylation site was also identified in purified proteins produced from insect cells, but not in proteins from *E. coli*. Although BRD4 has been reported to be an atypical protein kinase with auto-phosphorylation activity^30^, we did not observe any phospho-adducts in the bacterial samples by mass spectrometry and we did not detect any auto-phosphorylation of BRD4 by ADPglo assay in the presence of ATP (Supplementary Fig. 3).

Analysis by analytical size-exclusion chromatography (SEC) revealed differences in elution profiles that were construct and phosphorylation dependent: while phosphorylated BRD4^1–530^ eluted later than the unphosphorylated form, indicating protein compaction, the peaks of both phosphorylated BRD4^1–579^ and BRD4^1–722^ shifted toward earlier elution volumes, suggesting a phosphorylation-dependent oligomerization or structural elongation (Fig. 1c). Interestingly, the addition of high salt abrogated the effects of phosphorylation on BRD4^1–530^ but only had a partial effect on BRD4^1–579^ and no effect on BRD4^1–722^. Since ionic strength is known to modulate electrostatic interactions, these seem to predominantly drive the conformational compaction of BRD4^1–530^, while for the BRD4^1–579^ and BRD4^1–722^ structural changes, the electrostatic contributions are less sensitive to salt concentration and suggest that these interactions are stronger and may also involve additional hydrophobic contacts.

To further investigate the oligomeric state of the BRD4 constructs, size-exclusion chromatography multi-angle light-scattering (SEC-MALS), and analytical ultracentrifugation (AUC) sedimentation velocity and equilibrium experiments were undertaken (Fig. 1d and Table 1). First, in agreement with the earlier SEC experiments, both phosphorylated and unphosphorylated BRD4^1–530^ were monomeric, and the frictional coefficient (f/f_0_) and sedimentation coefficient both indicated that the phosphorylation resulted in a more compact form of the protein. Second, BRD4^1–579^ and BRD4^1–722^ samples were dimeric when phosphorylated and monomeric when unphosphorylated. Analysis of the frictional ratios and sedimentation coefficients also revealed interesting differences in the conformation of dimeric BRD4^1–579^ and BRD4^1–722^. The BRD4^1–722^ dimer adopted a more compact conformation than the truncated BRD4^1–579^ dimer, suggesting that the region comprising residues 580-722 mediates interactions that stabilize the compact conformation of BRD4^1–722^. The presence of the acetylation in the insect cell construct did not seem to affect the structure of the protein, as indicated by the similar behavior of CK2 in vitro phosphorylated proteins and insect cell samples.

**Table 1:** Summary of the SEC-MALS and AUC sedimentation velocity and equilibrium experiments.

BRD4 construct	State	Theoretical mass (Da)	Mass SEC-MALS (Da)	Equilibrium mass AUC (Da)	Average sedimentation coefficient (S)	f/f_0_*	Mass estimate
1-530	unphos. (bacteria)	60,512.7	81,330	62,883	2.58	2.35	64,200
	insect	60,569.7	86,970	68,124	3.18	2.10	79,500
	CK2 phos.	60,512.7	79,600	65,609	3.10	1.73	59,500
1-579	unphos. (bacteria)	66,376.5	87,130	80,511	3.13	1.97	86,100
	insect	66,433.5	129,500	91,765	4.42	2.40	152,000
	CK2 phos.	66,376.5	131,510	113,385	4.35	2.19	142,000
1-722	unphos. (bacteria)	82,415.3	96,170	117,258	3.53	1.72	78,200
	insect	82,472.3	147,200	145,923	5.65	1.58	123,000
	CK2 phos.	82,415.3	156,800	131,824	5.41	1.56	112,000
	7A CK2 phos.	82,303.3	105,200	–	–	–	–
	6A CK2 phos.	82,319.3	134,600	–	–	–	–
	Δ506-530 insect	80,404.9	91,200	–	3.71	1.76	80000
	Δ506-530 λ-phosphatase	80,404.9	81,750	–	2.95	2.41	82700
1-722 λ-phosphatase	+ iBET	82,472.3	88,157	–	–	–	–
	+ AZD5153	82,472.3	84,003	–	–	–	–
	+ 10	82,472.3	83,858	–	–	–	–
	+ 6	82,472.3	84,747	–	–	–	–
	+ 7	82,472.3	86,097	–	–	–	–
1-722 insect	+ iBET	80,404.9	120,321	–	–	–	–
	+ AZD5153	80,404.9	111,703	–	–	–	–
	+ 10	80,404.9	114,276	–	–	–	–
	+ 6	80,404.9	112,726	–	–	–	–
	+ 7	80,404.9	117,168	–	–	–	–

^*^f/f_0=_ frictional coefficient.

Overall, this analysis suggests that phosphorylation by CK2 induces a dimerization of BRD4 isoform C that is mediated by electrostatic and hydrophobic effects and is dependent upon the presence of the positively charged region BID.

### Motif B is involved in the phospho-driven structural change of BRD4

To gain insight into the structural rearrangements that occur upon phosphorylation, we compared the unphosphorylated and in vitro phosphorylated BRD4 constructs by Hydrogen-Deuterium eXchange Mass Spectrometry (HDX-MS). For all constructs, we identified a high number of unique peptides, common between the two phosphorylated states, which provided good coverage of BD1, BD2 (≥92%), and ET domains (74.7%) (Supplementary Fig. 4). Coverage of some parts of the BD1-BD2 linker and the BID region was not obtained, most probably due to the hydrophobic or highly positively charged primary sequences. Peptides derived from the NPS and CPS regions were excluded from the analysis, as these were differentially modified in the phosphorylated and unphosphorylated forms.

**Fig. 2:**
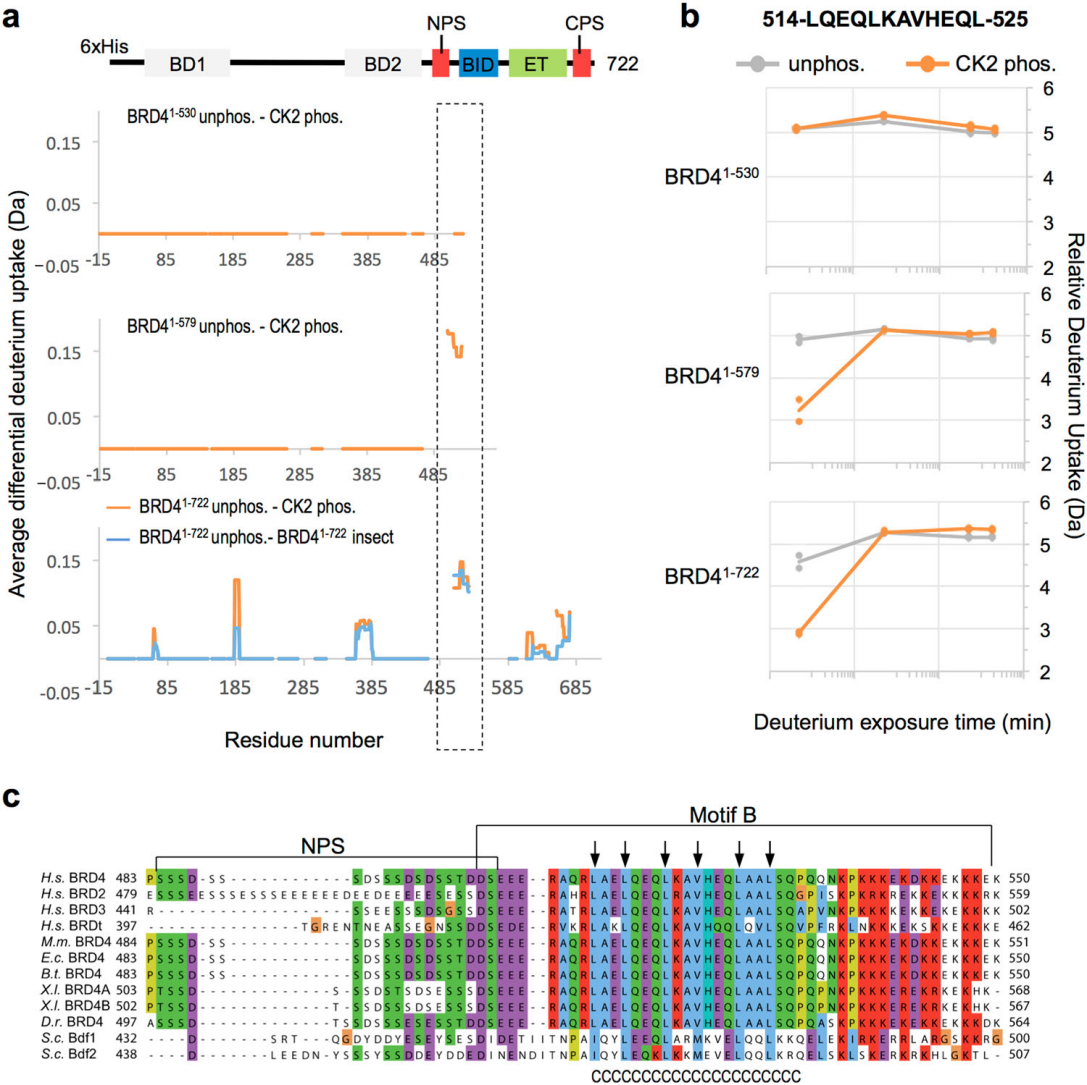
Change in Hydrogen-Deuterium exchange of motif B upon BRD4 dimerization. **a** Average difference of deuterium uptake for each residue of the indicated constructs (BRD4^1–530^, BRD4^1–579^, and BRD^1–722^) between the unphosphorylated sample (unphos.) and the sample subjected to CK2 phosphorylation (CK2 phos.) or purified from insect cell (insect). A positive differential uptake indicates a higher deuterium uptake (more exposed region) in the unphosphorylated sample and a protected region in the phosphorylated sample. Only peptides with changes above 0.5 Da and greater than 2.3x SD were taken into account. Areas with no coverage are represented as gaps. The protection upon phosphorylation of motif B observed in BRD4^1–579^ and BRD4^1–722^, but not in BRD4^1–530^, is highlighted with a rectangle. In these graphs, regions showing EX1 kinetics are considered with no difference. For details on the calculation, see the Materials and Methods section. Orange lines indicate the difference in deuterium update between unphosphorylated BRD4 1-722 and BRD4 1-722 phosphorylated by CK2 in vitro. Blue lines indicate the difference in deuterium update between unphosphorylated BRD4^1–722^ and insect cell-expressed BRD4^1–722^, **b** Example of a peptide in the region spanning aa 506-527 showing notable deuterium uptake differences over the shortest exposure time (3 sec on ice) upon CK2 phosphorylation in BRD4^1–579^ and BRD4^1–722^, but not in BRD4^1–530^. The relative deuterium uptake (Da) over deuterium exposure time is reported for each *n* = 3 independent experiments. Orange lines indicate CK2 phosphorylated samples and grey lines indicate unphosphorylated samples. **c** Sequence alignment of human BET proteins and BRD4 proteins from various species performed with ClustalOmega and represented with ClustalW color scheme. *H.s*.: *Homo sapiens*; *M.m*.: *Mus musculus*; *E.c.: Equus Caballus*; *B.t.: Bos taurus*; *X.l.: Xenopus laevis*; *D.r*.: *Danio rerio*; *S.c*.: *Saccharomyces cerevisiae*. The conserved NPS region with consensus CK2 phosphorylation sites (S/TxxE/D, where x is any residue) and Motif B are indicated. The JPred secondary structure prediction is reported below the sequences where residues predicted to be in a coiled coil are indicated below by ‘C’. Arrows point towards the conserved hydrophobic residues within the coiled-coil region that are predicted to form the inner hydrophobic interaction surface.

The largest change in deuterium incorporation was found in 6 overlapping peptides in BRD4^1–579^ and in BRD4^1–722^ spanning the region between the NPS and BID (aa 506-527) (Fig. 2a, b and Supplementary Fig. 4). Notably, these peptides exhibit reduced HDX in the phosphorylated proteins compared to the unphosphorylated counterparts, but this only occurred in BRD4^1–579^ and BRD4^1–722^, the two constructs that dimerize upon phosphorylation. In contrast, there was no change in deuterium uptake in the corresponding residues of BRD4^1–530^. The 506-527 region contains three heptad repeats, a characteristic of coiled-coil structures (Fig. 2c). Analysis of the BRD4 sequence using the LOGICOIL coiled-coil prediction algorithm^31^, strongly predicts a coiled-coil structure involving residues 506-527 with an antiparallel dimer configuration (Supplementary Fig. 5). Moreover, residues 506-527 are part of a region, named “motif B”, conserved among BET proteins (Fig. 2c) that has been proposed to mediate dimerization of BRD2 and other BET proteins, based on yeast 2-hybrid and co-immunoprecipitation data^32^.

Minor reductions in HDX were also observed in three other regions of phosphorylated BRD4^1–722^: aa 65–71 of the helix Z of BD1, aa 184–190 of the linker region immediately downstream of BD1, aa 362–386, which are part of the helix Z and the ZA-loop of BD2, and aa 621–644 and 657–675, which comprise most of the ET domain (Fig. 2a and Supplementary Fig. 6).

All of the HDX changes described above exhibit EX2 kinetics, which occur when the rate of protein refolding from a temporarily unfolded state is much faster than the rate of HDX, resulting in a gradual exchange of hydrogen. On the other hand, if the rate of protein refolding is slower than the rate of HDX, some residues will exchange before the protein returns to the folded state. In the HDX-MS analysis of all protein batches of BRD4^1–530^ and BRD4^1–722^, we identified peptides from the BD2 domain that displayed EX1 kinetics. This was not observed in the phosphorylated forms or in BRD4^1–579^ (Supplementary Fig. 7). The presence of EX1 kinetics suggests that BD2 has a more plastic structure than BD1, and that BD2 may be stabilized in the context of the BRD4^1–579^ construct or by the phosphorylation-driven conformational rearrangement of BRD4^1–530^ and BRD4^1–722^.

### Phosphorylation of BRD4 brings BD, BID, and ET regions in proximity

**Fig. 3:**
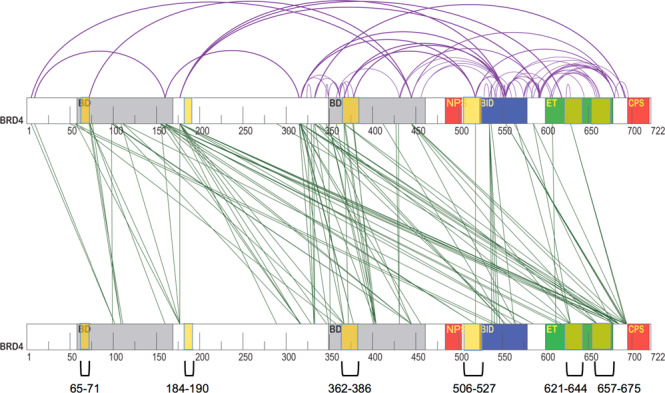
XL-MS analysis provides insight into the overall topology of the BRD4 dimer. Graphical representation of the cross-link (XL)-MS analysis. Cross-links found only in the CK2 phosphorylated sample (green) or only in the unphosphorylated sample (purple) are represented as inter-molecular cross-links or intra-molecular cross-links, respectively. The regions showing a reduced deuterium uptake upon phosphorylation in the HDX-MS experiment are highlighted with pale yellow: aa 65–71, 184–190, 362–386, 506–527, 621–644, 657–675.

To gain further insight into the architecture of the BRD4 dimer, we performed chemical crosslinking followed by MS (XL-MS) of the most relevant physiological form, BRD4^1–722^. When preparing cross-linked samples, a distinct slower-migrating band with a MW consistent with a dimer (165 kDa) appeared in both unphosphorylated and CK2 in vitro phosphorylated BRD4^1–722^ samples at increasing concentrations of cross-linker (Supplementary Fig. 8). The accumulation of the dimeric band was clearly more marked in the phosphorylated sample. This is consistent with BRD4^1–722^ having a propensity to form a dimer via the coiled-coil motif B that is stabilized only upon phosphorylation, thus resulting in the dimer being the main species present in solution for phosphorylated BRD4^1–722^. The unphosphorylated monomer and the phosphorylated dimeric species were purified by SEC prior to the analysis of the cross-linked peptides. In total, 69 putative intra-molecular cross-links and 104 inter-molecular cross-links were found, unique to the monomer or dimer, respectively (Fig. 3). These were mainly within or in the proximity of the BD1, BD2, BID, and ET regions, which suggests extensive cross-talk between the domains in the monomer that is increased after CK2 phosphorylation. Many putative inter-molecular cross-links were found between the C-terminal part of one molecule (BID and ET domain) and the N-terminus of the other molecule (BD1 and residues immediately downstream of it), suggesting a possible antiparallel arrangement of the BRD4^1–722^ dimer, in line with the LOGICOIL prediction of the coiled-coil oligomeric state (Supplementary Fig. 5). Interestingly, we found a putative inter-BRD4^1–722^ cross-link between the same lysine 519, located in the middle of the predicted coiled coil of motif B.

In general, we observed an agreement between the regions involved in putative inter-molecular cross-links and those with reduced deuterium uptake upon CK2 phosphorylation of BRD4^1–722^ in the HDX-MS analysis (Fig. 2a and Fig. 3).

### Aa 506-530 of motif B and the phosphorylation of NPS are required for BRD4 dimerization

**Fig. 4:**
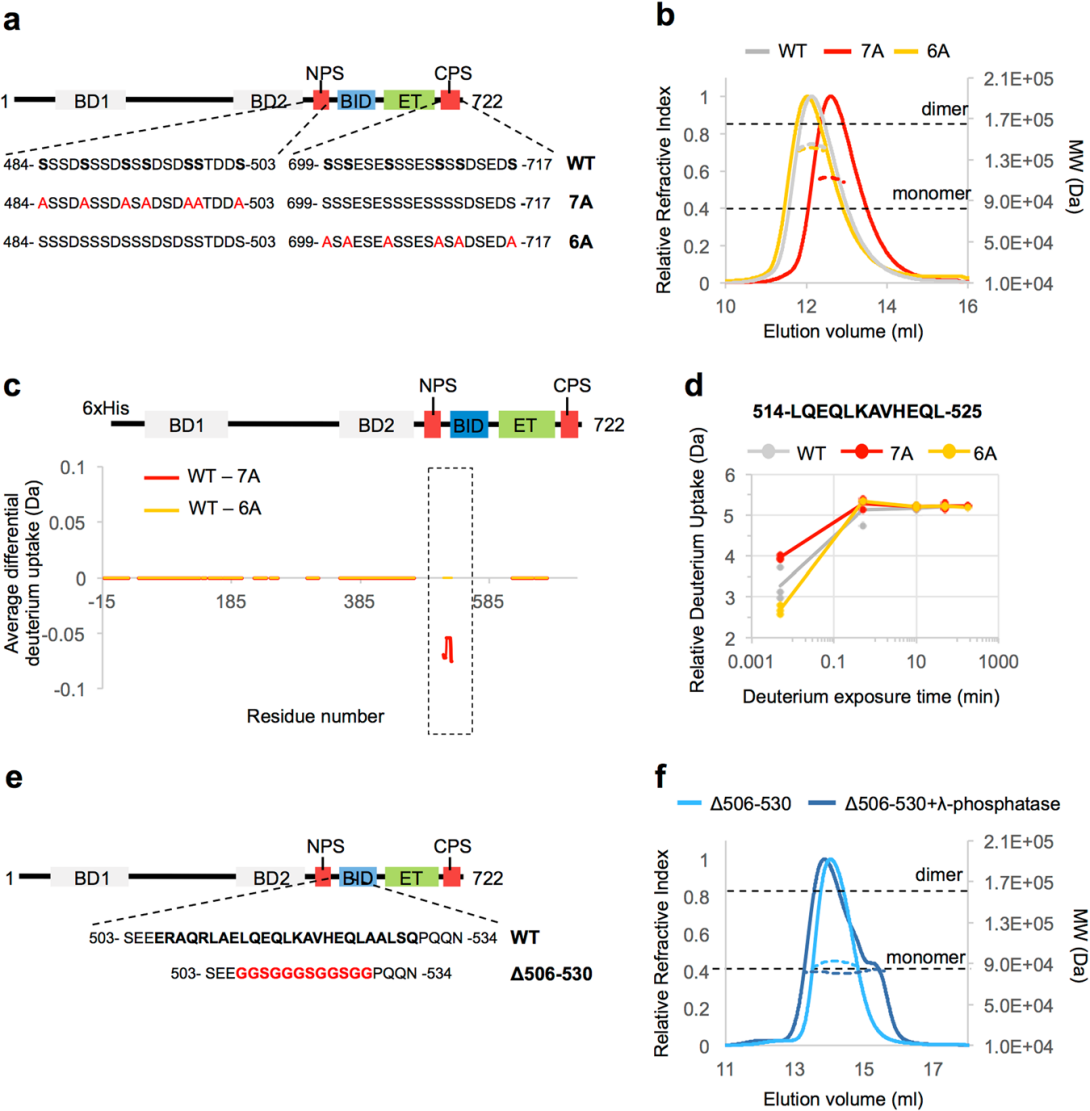
Biophysical analysis of the regions and the phosphorylation sites required for BRD4 dimerization. **a** Schematic overview of the BRD4^1–722^ phospho-deficient mutants 7A and 6A. In the wild-type (WT) sequence of NPS and CPS, the CK2 consensus sites are highlighted in bold. In the 7A and 6A mutant sequences, the serine residues mutated to alanine are highlighted in red. **b** SEC-MALS elution profiles for the BRD4^1–722^ constructs, produced in *E. coli* and phosphorylated by CK2. The grey line indicates WT, red line indicates the 7A mutant, and yellow line the 6A mutant. The dotted lines at each peak, colored as above, indicate the experimentally calculated MW. The dotted horizontal black lines represents the theoretical MW of the monomer or dimer of BRD4^1–722^, calculated from the wild-type primary sequence. **c** Difference of deuterium uptake for each residue between the WT and the 7A (in red) or 6A (in yellow) mutants. All samples have been produced in bacteria and phosphorylated by CK2. A negative differential uptake indicates a lower deuterium uptake (more protected region) in the wild-type sample. Only peptides with changes above 0.5 Da and greater than 2.3x SD were taken into account. The increased exposure of motif B in the 7A mutant is highlighted with a rectangle with dotted line. Areas with no coverage are represented as gaps. For details on the calculation, see the Materials and Methods section. **d** Example of the relative deuterium uptake of a peptide within motif B. The uptake is substantially reduced in the shortest exposure time (3 sec on ice) in the phosphorylated BRD4^1–722^ 7A mutant compared to the phosphorylated wild type. The relative deuterium uptake (Da) over deuterium exposure time is reported for each *n* = 3 independent experiments. Grey circles and line indicates WT, red circle and line 7A mutant, yellow circle and line 6A mutant. **e** Schematic representation of the BRD4^1–722^ ∆506-530 produced in insect cells. In the wild-type (WT) sequence, the region spanning residues 506–530 is in bold. In the ∆506–530 mutant sequence, the 12-residue glycine-serine-rich flexible linker used to replace the 506-530 aa sequence is highlighted in red. **f** SEC–MALS elution profiles of the BRD4^1–722^ ∆506–530 produced in insect cells and treated (blue line) or not treated (light blue line) with λ-phosphatase. The dotted line at each peak, colored as above, indicates the experimentally calculated MW. The dotted horizontal lines represent the theoretical MW of the monomer or dimer of BRD4^1-722^∆506–530.

Next, we sought to dissect the contribution of the two CK2 phosphorylated regions, NPS and CPS, to the structural rearrangement of BRD4^1–722^. To this end, we produced two mutant constructs containing serine-to-alanine mutations of all CK2 consensus sites in the NPS region (BRD4^1–722^ 7A) or in the CPS region (BRD4^1–722^ 6A) (Fig. 4a). After incubation with CK2, the expected reduction in phosphorylation of both the mutants relative to wild type was confirmed by mass spectrometry (Supplementary Fig. 1). Analysis by SEC-MALS revealed that the BRD4^1–722^ 6A mutant still displayed an apparent molecular weight similar to that of the wild-type protein and consistent with a dimer, while the behavior of the BRD4^1–722^ 7A mutant was consistent with monomers indicating that the CK2 sites of the NPS region, but not of CPS, are critical for phospho-dependent modulation of oligomeric state (Fig. 4b and Table 1). Moreover, comparison of the HDX uptake of the mutants to the wild-type protein, showed that the biggest difference was within motif B (aa 514–527), where HDX was lower in the BRD4^1–722^ 7A mutant, but unchanged in the BRD4^1–722^ 6A mutant (Fig. 4c, d). The minor HDX changes observed in BD1 and BD2, aa 184–190, and the ET domain may be due to phosphorylation of residues other than the consensus CK2 sites in the NPS and CPS. Taken together, these results indicate that phosphorylation of the NPS is required for the formation of the BRD4^1–722^ dimer. (Fig. 2a).

To test the role of motif B in the phospho-driven dimerization of BRD4^1–722^ we then produced mutant constructs in which residues 506-530 of BRD4^1–722^ were deleted and replaced with a 12-aa glycine-serine rich flexible linker (Fig. 4e). *E. coli* expression of BRD4^1–722^(Δ506-530) was greatly reduced suggesting that deletion of the coiled-coil region impacted protein stability; however, insect cell expression enabled purification of the samples. We then generated the unphosphorylated protein by incubating the insect cell BRD4^1–722^(Δ506–530) with λ-phosphatase. Mass spectrometry confirmed a substantial reduction in phosphorylation levels (Supplementary Fig. 9). Comparison of the oligomerization state of the phosphorylated and λ-phosphatase-treated BRD4^1–722^(Δ506–530) by SEC-MALS and AUC revealed that both the samples were present in monomeric state in solution (Fig. 4f, Table 1). Interestingly, the analysis of the frictional and sedimentation coefficients indicates a large conformational difference between the two samples, suggesting a more compact shape of BRD4^1–722^(Δ506-530) when phosphorylated (Table 1). This molecular rearrangement resembles the one observed for the BRD4^1–722^ dimer and indicates that phosphorylation is triggering a molecular compaction regardless of the change in oligomerization.

In summary, the Δ506–530 mutant protein analysis confirms that motif B is required for BRD4 dimerization and that the formation of the coil-coiled interface is essential for the stability of the BRD4^1–722^ dimer.

### BRD4 dimerization is detected in HCT116 cells by NanoBRET

**Fig. 5:**
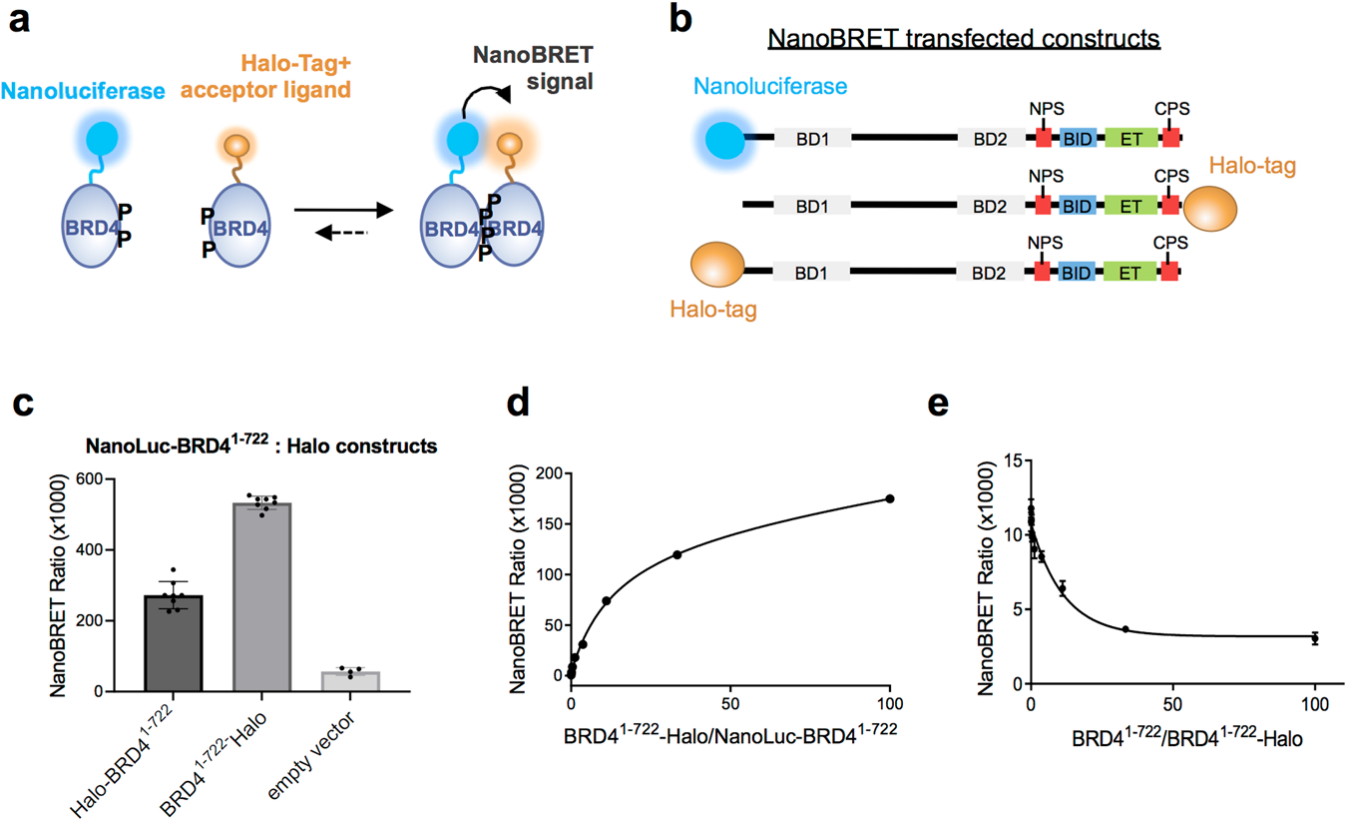
NanoBRET analysis of BRD4 dimerization in cells. **a** Schematic summary of the NanoBRET assays to test BRD4 homo-dimerization in HCT116 cells. The curved black arrow represents Bioluminescence Resonance Energy Transfer (BRET) between the Nanoluciferase donor (blue circle) and the Halo-tag acceptor ligand (orange circle). **b** Overview of the NanoBRET transfected constructs used in the NanoBRET experiments, with BRD4^1–722^ tagged at the N-terminus with Nanoluciferase, and at the N-terminus or at the C-terminus with Halo-tag. **c** NanoBRET signal observed using NanoLuc-BRD4^1–722^ and Halo-BRD4^1–722^ (dark grey bar) or NanoLuc-BRD4^1–722^ and BRD4^1–722^-Halo (grey bar). The single measurements and the mean with SD are reported (*n* = 8 for samples and *n* = 4 for control independent experiments). **d** Titration NanoBRET experiments where increasing amounts of acceptor DNA (BRD4^1–722^-Halo) were transfected with a fixed amount of donor DNA (NanoLuc-BRD4^1–722^). The mean with the standard error of the mean (SEM) is reported (*n* = 4 independent experiments) **e** Competition NanoBRET experiments where increasing amount of untagged BRD4^1–722^ DNA were transfected with a fixed amount of donor and acceptor DNA pair (NanoLuc-BRD4^1–722^ and BRD4^1–722^-Halo). The reduction of the NanoBRET signal at increasing values of untagged/Halo-tagged BRD4^1–722^ ratio indicates a specific competition of the untagged protein towards BRD4^1–722^-Halo for binding to NanoLuc-BRD4^1–722^. The mean with SEM is reported (*n* = 4 independent experiments).

The results of the in vitro biophysical analysis prompted us to test BRD4 dimerization in human cancer cells. We performed bioluminescence resonance energy transfer (NanoBRET) in HCT116 cells by transiently expressing two constructs of BRD4^1–722^ fused to either a Nanoluciferase tag (NanoLuc, donor) or a Halo-tag (acceptor) (Fig. 5a). A NanoBRET signal was observed in the presence of the BRD4^1–722^ NanoBRET pair (Fig. 5b, c), in contrast to control (Halo-tag only and NanoLuc-BRD4^1–722^) indicating that dimerization of BRD4^1–722^ occurs in this cellular context. It is worth to note that a higher NanoBRET signal was observed when the NanoLuciferase and the Halo-tag were added at the N-ter and C-ter of the molecules, respectively, consistent with an antiparallel arrangement of the dimer. The specificity of BRD4^1–722^ dimerization was confirmed in a saturation binding experiment with increasing concentrations of acceptor expression plasmid DNA (Fig. 5d), and in a competition experiment, in which untagged BRD4^1–722^ expression plasmid DNA was titrated against a constant amount of BRD4^1–722^ acceptor/donor pair plasmid DNA, leading to a reduction of the NanoBRET signal (Fig. 5e).

These results support the biophysical driven hypothesis that BRD4 dimerizes in cells.

### Effects of the binding of bivalent BET inhibitors to BRD4 conformation

**Fig. 6:**
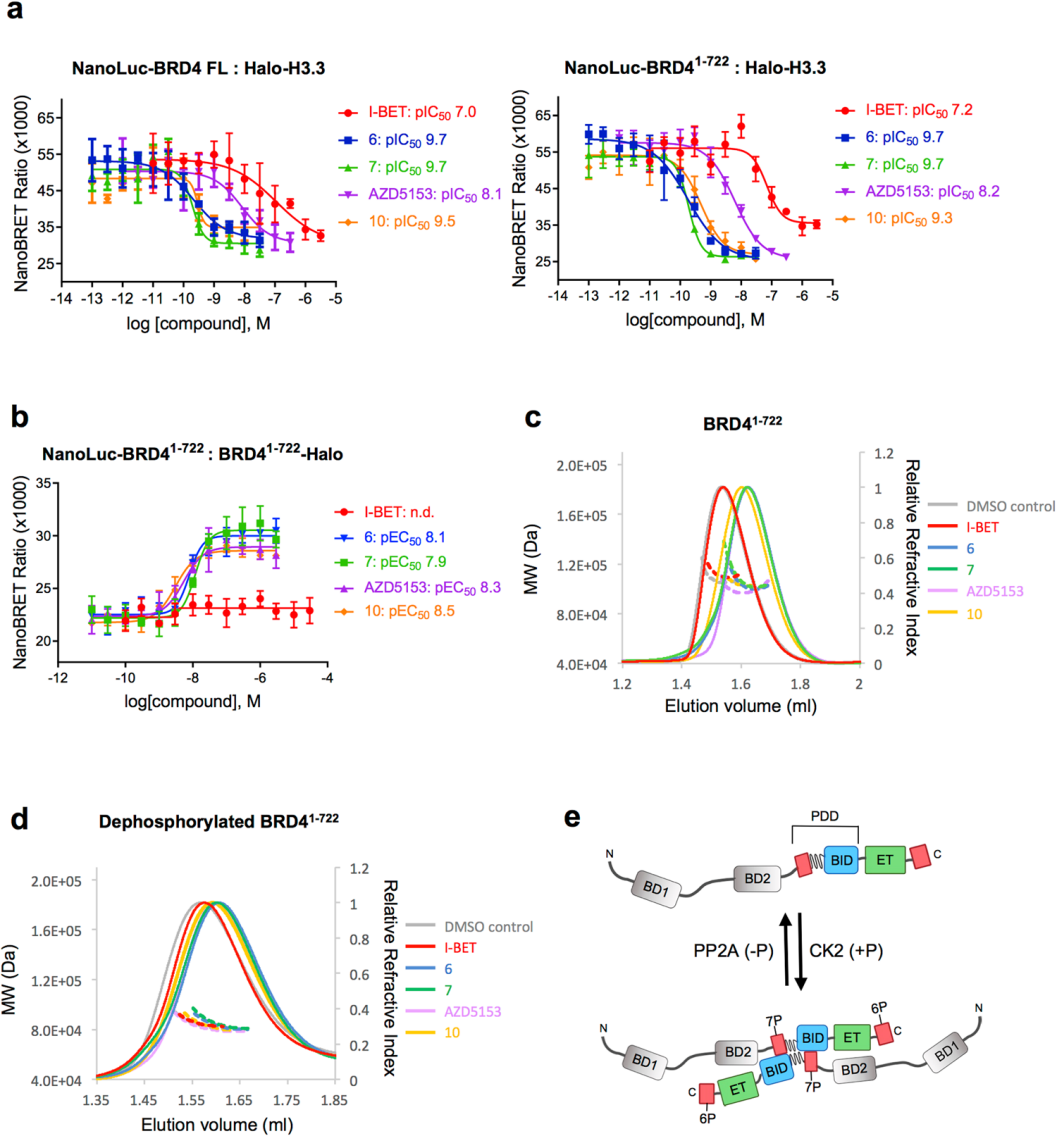
biBET inhibitors induce BRD4 compaction in vitro and in cells. **a** Effects of increasing concentration of bivalent compounds versus monovalent I-BET on the interaction between H3 and BRD4 full length or H3 and BRD4^1–722^ measured by NanoBRET. The mean with SD is reported (*n* = 4 independent experiments). **b** Effects of increasing concentrations of bivalent compounds versus monovalent I-BET on BRD4^1–722^ dimerization. The mean with SD is reported (n=4 independent experiments). **c** Effects of the addition of biBET inhibitors on the SEC-MALS elution profiles of BRD4^1–722^ produced in insect cells (phosphorylated) or **d** produced in insect cells and treated with λ-phosphatase (dephosphorylated). **e** Model of BRD4 dimerization driven by CK2 phosphorylation. The isoform C of BRD4, used in the study, is depicted. The NPS and CPS regions are represented as red boxes, the coiled-coil region (aa 506-530) is drawn as a wavy line and the phosphorylation-dependent dimerization domain (PDD) comprising NPS, the coiled-coil region and BID, is highlighted. The proposed conformation of the dimer is head to tail. Compounds are labeled as follows: I-BET red-circle and line; compound six blue square and line, compound seven green triangle and line, AZD5153 purple inverted triangle and line, compound 10 orange rhombus and line, DMSO control grey line.

We recently described a series of highly potent biBETs that simultaneously bind tandem bromodomains^23,33^ (Supplementary Fig. 10). These compounds were found to efficiently displace both BRD4 isoform A (1–1362) (FL) and isoform C (1–722) from histone H3 in NanoBRET assays with a much lower IC_50_ than the monovalent I-BET (Fig. 6a). We asked whether modulation of BRD4 oligomeric state might be relevant in the mechanism by which biBETs achieve exceptional potency in cell assays. Using our NanoBRET assay, we observed a concentration-dependent increase in BRD4^1–722^ BRET signal after addition of bivalent compounds, which was not detected with the monovalent I-BET (Fig. 6b**)**, reflecting either an increase in oligomerization or a conformational change that brings the NanoBRET donor and acceptor into closer proximity. Interestingly, for all but one of the bivalent compounds, the EC_50_ is >10-fold higher than the IC_50_ required to inhibit BRD4^1-722-^H3 binding. AZD5153 is the exception, which may suggest a different mode of inhibition relative to the other bivalent compounds. In order to differentiate between an increased oligomerization or a conformational change induced by iBETs, we performed SEC-MALS experiments on BRD4^1–722^ in the presence of compounds (Fig. 6c, d). We tested both the insect cell-expressed BRD4^1–722^, and its dephosphorylated monomeric form. Although SEC-MALS indicates a mixed population of dimer and monomer BRD4^1–722^ before dephosphorylation, all biBETs shift the BRD4 peak to a faster migrating population without change in molecular weight, whereas I-BET failed to shift the peak. The same was observed for the dephosphorylated BRD4 monomer. Our results indicate that biBETs induce conformational compaction in BRD4 with no effect on oligomeric state. This is consistent with previously observed biBET induced compaction of BRD4^44–460^ (BD1-BD2 tandem domain) in SAXS and AUC studies^23^.

These data suggest that biBETs employ the same binding mode toward monomeric and dimeric BRD4 and confirm that the conformational compaction of BRD4 is relevant to the mechanism of biBETs inhibition.

## Discussion

In this study, we provide a comprehensive biophysical and structural analysis of a series of constructs of human BRD4, including BRD4^1–722^, which represents the isoform C. Using SEC-MALS and AUC, we observed that BRD4^1–579^ and BRD4^1–722^, but not BRD4^1–530^, which lacks the BID domain, dimerize after phosphorylation by CK2. Dimerization of BRD4^1–722^ was also observed in mammalian cancer cells using NanoBRET assays. Based on the increased protection identified by HDX-MS, and on our mutation studies, we demonstrated that BRD4^1–722^ dimerization requires a coiled-coil region (aa 506-530) within motif B, BID, and phosphorylation of NPS. We propose a model for the phosphorylation-driven conformational change of BRD4 (Fig. 6e). In this model, unphosphorylated BRD4 is a monomer; however, upon phosphorylation by CK2, BRD4 forms a stable homodimer through an interface comprising the phosphorylated NPS, coiled-coil motif B and BID. Guided by I) the overall topology of the dimer obtained by XL-MS, II) the coiled-coil oligomeric state prediction by LOGICOIL and III) the previous observation with isolated domains that phosphorylated NPS binds to BID^29^, we propose a head to tail conformation of the dimer. In this conformation, the negatively charged phosphorylated NPS of one monomer contacts the positively charged BID of the other monomer, thus stabilizing the coiled-coil interaction. Although our data are based on the analysis of the BRD4 isoform C, the same phospho-regulated dimerization can be envisioned also for the long isoform A, which shares the same residues, including the regions forming the dimer interface. The biophysical and structural analysis of phosphorylated BRD4^1–722^ also suggests a compact shape for the dimer in which the bromodomains and the ET domain are in proximity to each other. This configuration would therefore create a multi-valent platform that brings protein ligands of BD2 and ET to the chromatin. Additional structural studies employing cryo-EM analysis of BRD4 in complex with binding partners and chromatin will be necessary to confirm this hypothesis.

The conformational phospho-switch model of BRD4 proposed by Wu et al.^1^ is based on an autoinhibitory activity of NPS toward BD1 and BD2, released by its phosphorylation and intra-molecular interaction with BID^29^. In our HDX-MS analysis, we did not observe an increase of HDX in BD1 or BD2 upon phosphorylation, as would be expected from the phospho-switch model due to release of autoinhibitory interactions. On the contrary, we observed a small HDX reduction in several peptides of BD1 and BD2, but only in BRD4^1–722^. Interestingly, the peptides of BD2 with reduced HDX are located in the ZA-loop and involve the WPF (W374 P375 F376) shelf important for creating the hydrophobic pocket hosting the acetylated lysines (Supplementary Fig. 6). The intra-molecular interactions, established upon BRD4^1–722^ phosphorylation and dimerization, may therefore stabilize BD2 for binding to chromatin, positive transcriptional elongation factor b or transcription factors. This hypothesis is further supported by the EX1 kinetics of HDX observed in BD2, which suggests an inherent plasticity of this specific bromodomain, with greater propensity to unfold in the unphosphorylated state.

Using phospho-deficient mutants in either NPS (7A mutant) or CPS (6A mutant), we identified NPS as a regulator of BRD4 dimerization. We did not observe any effect of phosphorylation of CPS on BRD4 structure in our biophysical analyses (Fig. 4), however this does not rule out a role for CPS in modulating interactions with other co-regulatory proteins. We have therefore identified a phosphorylation-dependent dimerization domain (PDD) in BRD4, spanning residues 484–579 and comprising NPS, coiled-coil motif B, and BID regions (Fig. 6e).

The coiled-coil interface of the BRD4 dimer is located within motif B, a region that was shown to be required for BRD2-chromatin binding in mitosis and was proposed to be a universal dimerization motif in BET proteins^32^. Our structural, biophysical and cellular analysis on BRD4 not only supports this hypothesis but also shows that dimerization is modulated by CK2-mediated phosphorylation of NPS and suggests a dynamic equilibrium between monomer and dimer. The NPS region of BRD2 has an insertion of around 13 negatively charged residues relative to BRD4 and fewer CK2 consensus phosphorylation sites, perhaps suggesting that BRD2 dimerization may not require phosphorylation. Our AUC data also demonstrate that the isolated BRD4 bromodomains are monomeric, whereas BRD2 BD1 has been shown to form stable dimers^34^, indicating differences in the nature or affinity of the dimer contacts in BRD4 and BRD2.

It is interesting to note that many reported BRD4 binding partners are oligomers: p53 (tetramer) binding to BID^29^; viral latency-associated nuclear antigens kLANA and mLANA (both the dimers and higher oligomers) binding to ET^35,36^; histone H3 and H4 (two copies of which are included in the octameric nucleosome), binding to BD1 and BD2. Dimerization of BRD4, and the resulting spatial proximity of four bromodomains, two ET domains, and two BIDs, could therefore lead to the right architecture not only for binding to multiple acetylated histone tails of the same or adjacent nucleosome, but also for the efficient recruitment of the other interacting oligomers.

The BRD4 isoform C forms liquid-like phase separations (LLPS) at the nucleus involved in active gene transcription^37^ similar to those previously reported for the long isoform A. LLPS and DNA binding was inhibited by phosphorylation via CK2 and LLPS were also inhibited by the addition of a bivalent inhibitor, but not by JQ1. Phosphorylation of BRD4 therefore promotes dimerization and inhibits interaction with DNA and formation of LLPS, while being necessary for active gene transcription. The apparently conflicting effects of phosphorylation in promoting gene transcription^24^, while reducing LLPS in chromatin^32^, may suggest that phosphorylated and unphosphorylated BRD4 form different molecular associations in LLPS. The transient polyvalent self-associations of unphosphorylated BRD4 LLPS^32^ contrast with the stable dimeric interaction of phosphorylated BRD4. The level of BRD4 phosphorylation may therefore modulate the structural and ligand binding properties of the BRD4 LLPS to decrease DNA binding and enhance interaction with transcriptional complexes, while maintaining interaction with acetylated histones so that when levels of BRD4 phosphorylation rise above a certain threshold transcription is triggered at gene loci. This suggests that phosphorylation and dephosphorylation of BRD4 mediates a dynamic interplay between DNA binding and dimerization to regulate transcription.

In the last few years, potent and selective inhibitors targeting the bromodomains of BET proteins have been developed^38,39^. The biochemical and structural assays guiding drug design were mainly based on truncated constructs comprising BD1, BD2 or BD1-BD2. In BRD4, the finding that regions downstream of BD2 are involved in phospho-regulation of binding to chromatin and other interacting partners^27,29^, and in the development of resistance against iBETs^28^, underlines the need to employ more physiologically relevant constructs in biochemical and biophysical studies to better resemble the native context.

Our NanoBRET experiments in HCT116 cells and SEC-MALS data indicated a conformational change of BRD4^1–722^ upon addition of bivalent compounds, which target two bromodomains simultaneously (Fig. 6). We previously showed that biBETs afford an increase in potency in cell assays over corresponding mono-dentate inhibitors of up to four orders of magnitude and that biBETs are capable of engaging both bromodomains in BD1-BD2 constructs simultaneously^23^. Here, we confirm that biBETs are able to induce a protein rearrangement of BRD4, independently of its oligomeric state.

Our results show that phospho-dependent BRD4 dimerization brings BD2 and ET domains into proximity, suggesting that a bivalent strategy, similar to that of biBETs, might be a viable approach to simultaneously target BD and ET domains, thus influencing coregulator interactions and perhaps resulting in inhibitors with different pharmacological and safety profiles. The NMR-derived structure of the ET domain was recently solved with a peptide ligand suggesting the ET domain as a target for small-molecules inhibitors^40^. Bivalent strategies may be of wider value in targeting phase-separated condensates where the high concentrations of multi-domain proteins with flexible intrinsically disordered regions may provide enhanced avidity for binding.

In conclusion, our study provides an important contribution to the understanding of the molecular details of BRD4 function, and a refined model for BRD4 activation and inhibition that can be employed in drug discovery for the development of more specific, effective and safe therapies and suggests a general strategy for therapeutic targeting of epigenetics by exploiting avidity to achieve potency and selectivity in binding.

## Materials and methods

### Recombinant protein production

Variants of human BRD4 were generated by gene synthesis (GeneArt, Life Technologies) with an N-terminal 6xHistidine (6xHis) tag or 6xHis-Halo tag followed by tobacco etch virus (TEV) protease site. For bacterial expression, constructs were subsequently cloned into a pET28b vector and transformed into Escherichia coli (*E. coli*) Bl21 Gold (DE3) strain (Novagen). Protein expression was induced at 0.6–0.8 OD_600_ with 0.1 mM IPTG and sustained overnight at 18 °C. For insect cell expression, constructs were cloned into a pFastBac1 and bacmid DNA was produced in *E. coli* DH_10_ Bac cells. Recombinant baculoviruses were generated in Sf21 cells (Thermofisher) and protein expression was conducted at 27 °C for 48 h. Cells were lysed using a Constant Systems cell disruptor in 50 mM Hepes pH 8.0, 300 mM NaCl, 5 mM Tris(2-carboxyethyl) phosphine hydrochloride (TCEP), 10 mM Imidazole, 10% Glycerol, 1x Complete EDTA-free protease inhibitors (Roche) and benzonase nuclease (5 u/ml, Sigma). For insect cell samples, 1x Halt phosphatase inhibitors (Thermo Fisher Scientific) were additionally added. Cells were clarified by centrifugation at 43260 rcf for 45 min at 4 °C and incubated with Ni-NTA agarose (QIAGEN) overnight at 4 °C. Bound proteins were washed with lysis buffer supplemented with NaCl to 1 M and eluted in 50 mM Hepes pH 8.0, 50 mM NaCl, 1 mM TCEP, 300 mM Imidazole, 5% Glycerol. BRD4 was further purified by ion-exchange chromatography using Resource columns (GE Healthcare) and a 50-500 mM NaCl linear gradient. A final purification step by SEC was performed using HiLoad Superdex 200 16/600 column (GE Healthcare) in storage buffer (10 mM Tris pH 8.6, 500 mM NaCl, and 1 TCEP).

CK2α^1–335^ expression construct was synthesized untagged by GeneArt (Life Technologies) and cloned into a pET28b vector. Protein expression was conducted as described for BRD4 bacterial expression. Cells were lysed using a Constant Systems cell disruptor in 25 mM Tris pH 8.5, 300 mM NaCl, 1 mM TCEP, 1x Complete EDTA-free protease inhibitors (Roche), and benzonase nuclease (5 u/ml, Sigma). After centrifuging at 43260 rcf for 45 min at 4 °C, the supernatant was loaded onto a HiTrap Heparin column (GE Healthcare) and eluted with a 0.3–1 M NaCl linear gradient. Fractions containing CK2α^1–335^ were diluted to a final NaCl concentration of 100 mM, loaded onto Resource Q (GE Healthcare) and eluted with a 100–500 mM NaCl linear gradient. Protein fractions were further purified by SEC with a HiLoad 16/600 Superdex 75 column (GE Healthcare) in 25 mM Tris pH 8.5, 500 mM NaCl, 1 mM DTT.

### In vitro phosphorylation of BRD4 by CK2

During purification, ion-exchange fractions containing bacterial BRD4 constructs were pulled together and diluted 1:2 into a final reaction including 50 mM Tris pH 7.5, 10 mM MgCl_2_, 0.1 mM EDTA, 2 mM DTT, 500 µM ATP, 0.5x Complete EDTA-free protease inhibitors (Roche), 0.5x Halt phosphatase inhibitors (Thermo Fisher Scientific) and CK2α^1–335^ with a protein:kinase ratio of 15:1 (w:w). The reaction was incubated overnight at 4 °C, supplemented with 500 µM ATP and further incubated at 30 °C for 2 h. Phosphorylated BRD4 constructs were isolated by anion-exchange with a Resource Q column and further subjected to SEC using Superdex 200 10/300 GL column in storage buffer (10 mM Tris pH 8.6, 500 mM NaCl and 1 TCEP).

### Intact mass spectrometry analysis

Samples were desalted and concentrated with 0.5 ml Millipore Amicon Ultra cut-off filters (UFC505008, UFC503024) in a refrigerated centrifuge (4 °C). The mobile phase used for gradient elution consisted of (A) 0.1% formic acid (Fluka 5630-10XML-F) in water (JT Baker, 4218-02) and (B) 0.1% formic acid in Acetonitrile (JT Baker, JT9017-2). The LC/MS system used a Shimadzu Prominence HPLC with a Agilent C8 column (Poroshell StableBond 300 C8, 2.1 × 75 mm, 5 µm) at 500 ul/min flow rate with a gradient consisting of 1 min at 20% B, then ramp to 95% B over 4 min, then hold for 1 min at 95% B before returning to 20% B. Mass spectra (LC/MS) were acquired on a Sciex 5600 TripleTOF+ mass spectrometer (Foster City, CA) using Analyst 1.6 software (Foster City, CA). Source temperature was 450 C, spray voltage (ISVF) was 5500 V, curtain gas was 30, GS1 = 60, GS2 = 70, and data were acquired over 1000–4000 Da mass range. Protein peak reconstruction (charge state deconvolution) used the BioToolKit MicroApp v2.2 in the Sciex PeakView 2.2 software.

### ADPglo assay

Luminescent ADP detection assay was performed using the ADPglo kit (Promega). Eleven serial two-fold dilutions of BRD4^1–722^ starting from 85 µM were prepared in assay buffer (50 mM Tris pH 7.5, 10 mM MgCl_2_, 0.1 mM EDTA, 2 mM TCEP). For the reaction, 2 µl of each BRD4 dilution was mixed with 0.6 µM CK2α^1–335^ and 0.2 mM Ultra Pure ATP (Promega) in a final volume of 5 µl and incubated at RT for 1 h. The ADPglo reagents were added as described in the ADPglo kit protocol. Luminescence was quantified using the EnVision 2014 plate reader (Perkin Elmer) and analysed with Prism (GraphPad).

### Analytical SEC

For each protein sample, 25 µl at 20 µM was injected into a Superdex200 PC 3.2/30 column (GE Healthcare) equilibrated with 10 mM Tris pH 7.5, 250 mM NaCl, 1 mM TCEP. For high salt analysis, a buffer containing 10 mM Tris pH 7.5, 1 M NaCl, 1 mM TCEP was used.

### Analytical ultracentrifugation

All analytical ultracentrifugation experiments were performed on either a Beckman XLA or XLI ultracentrifuge. Sedimentation equilibrium experiments were performed using 6-sector cells with 110 ul of sample in 10 mM Tris-HCl pH 7.5 with either 250 mM or 1 M NaCl. Samples of between 0.1 µM and 15 μM were centrifuged at speeds of 9,000, 13,000, and 20,000 rpm for 15-hours where equilibrium was attained and scanned using wavelengths of 230 nm and 280 nm. Data was selected based on appropriate absorbance and speed and analysed using HeteroAnalysis developed by James Cole and Jeffrey Lary, Version 1.1.57 using a single species model and floating the buoyant molecular weight. Sedimentation velocity was used to ascertain the sedimentation coefficients in both 250 mM NaCl and 1 M NaCl. Sedimentation velocity experiments were performed using a An50Ti rotor and standard 2-sector Epon centerpieces and quartz windows. Samples were diluted prior to loading in the centrifuge and referenced with the corresponding buffer. The ultracentrifuge was run at a speed of 45,000 rpm collecting scans at 280 nm until full sedimentation had been reached. Samples were analysed using the program Sedfit developed by Peter Schuck^41^. Sedimentation coefficient distributions were corrected to standard conditions using the buffer density and viscosity correction. The partial specific volume was set to 0.73 cm3/g.

### SEC-MALS

In order to characterize BRD4 oligomeric state, size-exclusion chromatography coupled to multi-angle light scattering was used to ascertain the weight-average mass of particles eluting from a gel filtration column using a Wyatt Helios II 18-angle light-scattering instrument coupled to a T-Rex differential refractometer and a QELS in-line dynamic light-scattering instrument. A Superose 6 (GE-life sciences) 24-ml gel filtration column was used to separate proteins according to their molecular weight. The column was equilibrated in 10 mM Tris-HCl pH 7.5 with either 250 mM or 1 M NaCl and samples were loaded using a flow rate of 0.75 ml/minute on a Bio-Rad NGC FPLC instrument. The mass and polydispersity of the samples eluting from the column was performed using the angular dependence of the scattered light from the sample in the light-scattering detector, and the concentration from the differential refractive index detector. A value of 0.183 ml/g was used for the dn/dc value.

In order to characterize the effects of bivalent BET inhibitors on BRD4, SEC-MALS was performed on a Malvern Omnisec Resolve/Reveal system compromising of LALS, RALS, UV, RI, and a viscometer. Insect cell-expressed BRD4 (1-722), before and after dephosphorylation at a concentration of 2 mg/ml (25 μM) was mixed with compounds to a final concentration of 25 μM, 1% DMSO and incubated on ice for 1 h. For each sample, 10 µl was injected into a Superdex200 increase 3.2/300 column (GE Healthcare) equilibrated with 10 mM Tris pH 8, 300 mM NaCl, 2 mM TCEP.

### Hydrogen-deuterium exchange mass spectrometry (HDX-MS)

HDX-MS experiments were performed in triplicates for each time point. Protein stock solution concentrations were adjusted to 70 µM using BRD4 storage buffer (10 mM Tris pH 8.6, 500 mM NaCl, and 1 TCEP) and further diluted to 15 µM with dilution buffer (20 mM Tris pH 7.0, 150 mM NaCl, 1 mM TCEP) in order to reach a final NaCl concentration of 230 mM and a final pH of 7.5. Hydrogen-deuterium exchange reactions were conducted using the automated LEAP H/D-X PAL system (LEAP Technologies) as follows: 5 μl of BRD4 constructs at 15 µM were added to 50 µl D_2_O labeling buffer (20 mM Tris pD 7.5, 150 mM NaCl, 1 mM TCEP, and 94.8% D_2_O) at 20 °C. The hydrogen-deuterium exchange reactions were quenched at different time points (0.5, 50, 180 min) by transferring 50 μl of the mixture to 50 μl of pre-chilled quench solution (4 M Urea, 1.8% formic acid, pH 2.5) at 0 °C and 80 μl of the reaction+quenched solution was then injected into a Acquity UPLC M-class system (loop volume 50 μl)(Waters). Undeuterated sample (time point 0) was prepared identically, except substituting D_2_O labeling buffer with a H_2_O labeling buffer. For the shortest time point (0.3 sec), the reaction was manually prepared, by incubating 5 μl of BRD4 constructs at 15 µM with 50 μl D_2_O labeling buffer on ice for 3 s. After mixing 50 μl of reaction with 50 μl ice-cold quench solution, the sample was immediately snap-frozen in liquid nitrogen and kept at −80 °C until LC/MS analysis, prior to which, it was quickly thawed and injected into the UPLC system.

In order to measure deuterium incorporation the quenched samples were injected into an immobilized pepsin column (Enzymate BEH-Pepsin column, Waters) for 3 min at 20 °C with a flow rate of 100 µl/min of 95% buffer A (0.1 % formic acid in H_2_O) and 5% buffer B (0.1% formic acid in acetonitrile). Peptides were trapped on a VanGuard C18 Pre-column (Waters) and subsequently separated on a C18 reverse phase analytical column (100 mm × 1 mm, Waters) at 0.5 °C, using a gradient of 10–40% buffer B over 11 min at a flow rate of 40 μl/min. Eluents were analysed on a Synapt G2-Si (Waters), acquiring over a mass range of 50–2000 *m/z*, using an ESI source operated at 200 °C and a spray voltage of 3 kV. In order to avoid carryover, a blank was run after each sample by injecting buffer A, and two washes of the pepsin column were performed after each run with 1x Quench buffer + 20% methanol.

For peptide identification, the data for the undeuterated sample were acquired in MS^E^ mode and analysed with ProteinLynx Global Server (PLGS, Waters). For all samples and all-time points, peptides with at least 5000 intensity, 0.3 products per amino acids, 1 consecutive product, and a PLGS score of 6.4, were analysed using DynamX (Waters). The isotope peaks were identified automatically by the software and then manually validated in order to exclude ambiguous annotation or overlapping peptides. For every time point of every peptide, the deuterium uptake is calculated by subtracting the centroid of the peptide isotopic distribution at time 0 from the centroid of the peptide isotopic distribution at each time point. For each time point, only averages of deuterium uptake difference greater than 0.5 Da and higher than 2.3x standard deviation were considered significant. The deuterium incorporation per residue was calculated by taking into account the average deuterium uptake per residue of all overlapping peptides, as in Eq. (1):

1 $$re{s}_{j}=\frac{1}{N}\,\mathop{\sum }\limits_{i=1}^{N}\frac{pe{p}_{i}}{amid{e}_{i}}$$

$re{s}_{j}$: mean deuterium uptake difference of residue j.

$N$: number of overlapping peptides containing residue j.

$pe{p}_{i}$: deuterium uptake difference of peptide i, containing residue j.

$amid{e}_{i}$: number of exchanging residues of peptide i.

All results are presented as relative levels of deuterium incorporation where no correction for back exchange is applied. Peptides showing EX1 kinetics were analyzed with HX-Express2^42^.

### Cross-linking mass spectrometry (XL-MS)

Purified solutions of both unphosphorylated and CK2 in vitro phosphorylated BRD4^1–722^ were diluted to a concentration of 0.082 mg/ml in 20 mM HEPES pH 7.8, 250 mM NaCl, and 1 mM DTT and cross-linked using a homobifunctional, isotopically-coded N-HydroxySuccinimide (NHS) ester BS3 (H_12_/D_12_) purchased from Creative Moleucles (Canada) at a concentration of 0.058 mg/ml.

The cross-linked samples were fractionated by size-exclusion chromatography on a Superose 6 Increase 3.2/300 column with 20 mM HEPES pH 7.8, 250 mM NaCl and 1 mM DTT at a flow-rate of 50 µl/min and fraction collected every minute. Each fraction collected at elution volumes between 1.7 and 2.2 ml was checked by SDS-PAGE and the amount of both dimer and monomer evaluated. Fractions eluting between 2 and 2.2 ml, enriched in monomers, were combined for both unphosphorylated and CK2 in vitro phosphorylated BRD4^1–722^ samples. Dimers were mainly observed in fractions between 1.7 and 1.85 ml and were combined for CK2 in vitro phosphorylated BRD4^1–722^.

Cross-linked samples were freeze-dried and resuspended in NH_4_HCO_3_ 50 mM to a protein concentration of 1 mg/ml. The samples were reduced and alkylated with DTT 10 mM and iodoacetamide 50 mM, respectively. Proteins were sequentially digested with trypsin and Glu-C. Both the enzymes were added at an enzyme-to-substrate ratio of 1:20 and the reaction incubated overnight at 37 °C. The reaction was started by trypsin followed by Glu-C addition after 4 h. After digestion, the samples were acidified with formic acid and the peptides were fractionated by peptide size-exclusion chromatography.

Digests were then fractionated by peptide-level size-exclusion chromatography using a Superdex Peptide 3.2/300 (GE Healthcare) with a 30% Acetonitrile 0.1% TFA mobile phase at a flow rate of 50ul/min. Fractions were collected every 2 min from the elution volume 1.0 ml to 1.7 ml. Before LC-MS analysis fractions were dried and resuspended in 2% Acetonitrile and 2% formic acid.

The digests were analysed by nano-scale capillary LC-MS/MS using an Ultimate U3000 HPLC (ThermoScientific Dionex, San Jose, USA) to deliver a flow of approximately 300 nL/min. A C18 Acclaim PepMap100 5 µm, 100 µm × 20 mm nanoViper (ThermoScientific Dionex, San Jose, USA), trapped the peptides prior to separation on a C18 Acclaim PepMap100 3 µm, 75 µm × 250 mm nanoViper (ThermoScientific Dionex, San Jose, USA). Peptides were eluted with a gradient of acetonitrile. The analytical column outlet was directly interfaced via a nano-flow electrospray ionization source, with a hybrid dual pressure linear ion trap mass spectrometer (Orbitrap Velos, ThermoScientific, San Jose, USA). MS data were acquired in data-dependent mode. High-resolution full scans (*R* = 30,000, m/z 300-2000) were recorded in the Orbitrap. The ions correposnding to the 10 most intense MS peaks were sequentially selected and CID activated (normalized collisional energy 35). MS/MS scans were acquired in the linear ion trap.

Xcalibur raw files were converted into the MGF format using MSConvert (Proteowizard)^43^ and used directly as input files for Stavrox^44^. Searches were performed against the protein sequence and a set of randomized decoy sequences generated by the software. The following parameters were set for the searches: maximum number of missed cleavages 3; minimum and maximum peptide length of 5 and 10 amino acids, respectively; variable modifications: carbamidomethyl-Cys (mass shift 57.02146 Da), Met-oxidation (mass shift 15.99491 Da); cross-linker composition: C_8_H_10_O_2_ for H_12_-BS3 and C_8_H_10_O_2_D_12_-H_12_ for D_12_-BS3; residue pairs considered for cross-linking reaction: K-K, K-S, K-Y, K-T; MS1 tolerance 5 ppm, MS2 tolerance 0.5 Da; false discovery rate cut-off: 5%. The MS/MS spectra of identified cross-links were manually inspected and validated.

### NanoBRET

Plasmids for the NanoBRET experiments were constructed by subcloning BRD4^1–722^ constructs into N-terminally tagged NanoLuc-TEV (pFN31K) or C-terminally tagged Halo-TEV (pFC14K) vectors (Promega). Untagged BRD4^1–722^ construct was prepared by amplifying BRD4^1–722^ with STOP codon and cloning it into pFC14K vector. Halo-TEV-H3.1 in pFN21A was obtained from Promega. HCT116 human colorectal carcinoma cells (ATCC; CCL-1573) were cultured in McCoy’s 5 A medium containing 2 mM glutamine and 10% FCS. For transfection, 8 × 10^5^ cells were seeded into a 6-well culture plate and allowed to attach for 4 h. A mixture containing 2 µg of Halo-tagged protein vector, 0.02 µg of NanoLuc-tagged protein vector, and 8 µl Fugene HD (Promega) was added to each well. For the titration experiment, 4 × 10^5^ cells were seeded into a 12-well culture plate and allowed to attach for 4 h. Three-fold serial dilutions of Halo-tagged BRD4^1–722^ vector were prepared in 1 µg/µl transfection carrier DNA (herring sperm DNA, Sigma) starting from 1 µg/µl. One µl of each dilution was combined with 10 ng of NanoLuc-tagged protein vector and 4 µl Fugene HD and added to each well. Transfections for the competition experiment were performed as for the titration experiment with the following differences: 8x serial 3-fold dilutions of untagged BRD4^1–722^ vector were prepared in 1 µg/µl transfection carrier DNA (herring sperm DNA, Sigma) starting from 1 µg/µl; 1 µl of each dilution was combined with 10 ng of Halo-tagged BRD4^1–722^ vector, 1 ng of NanoLuc-tagged protein vector, and 4 µl Fugene HD, and added to each well of the 12-well culture plate. In all cases, proteins were allowed to express at 37 °C in 5% CO_2_ for approximately 20 h. Cells were then harvested and resuspended in OptiMeM (Life Technologies) with 4% fetal calf serum at 2 × 10^5^ cells/ml in presence of 100 nM Halo-Tag 618 Ligand (Promega) or 0.1% DMSO (control). Forty microliter (8000 cells) were transferred into a white, flat-bottomed, tissue-culture-treated 384-well plate (Greiner). When testing compounds, varying amounts solubilized in DMSO were previously added to the plate using an automated D300 Digital Dispenser (TECAN), normalizing the final DMSO concentration to a maximum of 0.3%. Plates were incubated for approximately 18 h at 37 °C in the presence of 5% CO_2_. NanoBRET Nano-Glo Substrate (Promega) was added to both control and experimental samples at a final concentration of 10 μM. Plates were read within 10 min using a Pherastar FS multimode plate reader (BMG Labtech) equipped with a NanoBRET filter module (excitation 450 nm, emission 610nm-LP). The results were reported as milliBRET units (acceptor emission value 610 nm/donor emission value 450 nm) × 1000). Data were fitted with Prism (GraphPad), using the following equations: one site-total non-linear equation for the titration experiment [Y = Bmax*X/(Kd+X) + NS*X, where Bmax is the maximum specific binding, Kd is the equilibrium binding constant, NS is the slope of nonspecific binding]; variable four-parameter curve fit for testing effects of the compounds on BRD4 dimerization or BRD4-H3 interaction [Y=Bottom + (Top-Bottom)/(1 + 10^((LogEC50-X)*HillSlope)^)]; variable four-parameter curve fit for testing the effects of the compounds on the BRD4-H3 interaction and for the BRD4 competition experiment [Y=Bottom + (Top-Bottom)/(1 + 10^((LogIC50-X)*HillSlope)^)].

### Statistics and reproducibility

The details about experimental design and statistics used in different data analyses performed in this study are given in the respective sections of results and methods. For the HDX-MS experiments using BRD4^1–530^, BRD4^1–579^, and BRD4^1–722^, different biological samples were analyzed comprising protein from different purification batches.

### Reporting summary

Further information on research design is available in the Nature Research Reporting Summary linked to this article.

## Supplementary information

Supplementary Information

Supplementary Data 1

Supplementary Data 2

Supplementary Data 3

Reporting Summary

## Data Availability

All relevant data are within the paper and its Supplementary Information file. The source data of the HDX-MS, XL-MS and NanoBRET experiments are provided as Source Data files. Uncropped SDS-PAGE gel images are provided in Supplementary Figure. Source data for the graphs and charts in the main figures is available as Supplementary Data 3. Additional data that support the findings of this study are available from the corresponding author on request.
