# Supplementary material for: Phosphorylation-dependent BRD4 dimerization and implications for therapeutic inhibition of BET family proteins
Source: Commun Biol. 2021 Nov 9;4:1273. doi: 10.1038/s42003-021-02750-6 (PMC8578508; doi:10.1038/s42003-021-02750-6)

Supplementary information

Phosphorylation-dependent BRD4 dimerization and implications for therapeutic inhibition of BET family proteins

Francesca Malvezzi, Christopher J. Stubbs, Thomas A. Jowitt, Ian L. Dale, Xieyang Guo, Jon P. DeGnore, Gianluca Degliesposti, J. Mark Skehel, Andrew J. Bannister, Mark McAlister

Supplementary Figure 1

Intact mass spectrometry of BRD4 constructs differentially phosphorylated. The phosphorylation state of $BRD4^{1-530}$, $BRD4^{1-579}$, $BRD4^{1-722}$, purified either from bacteria (bacteria) or insect cells (insect), or bacterially purified and then phosphorylated by CK2 (CK2 phos.) was analysed by mass spectrometry. The average molecular weight predicted from the primary sequence is reported. The number of phospho groups calculated based on the difference between observed and predicted molecular weight is shown next to the corresponding peak. In the constructs purified from insect cells, a parallel set of peaks is indicated, corresponding to the acetylated protein differentially phosphorylated (+42 Da difference). All figures were resized to 6” x 9.1”, 73%, with the following parameters: Resolution = 30000; Gaussian smoothed (2 points); Step = 0.5; Charge agent = H+; Deconvolution output mass range = +/- 2 kDa of expected average mass.

BRD4$^{1-530}$ bacteria

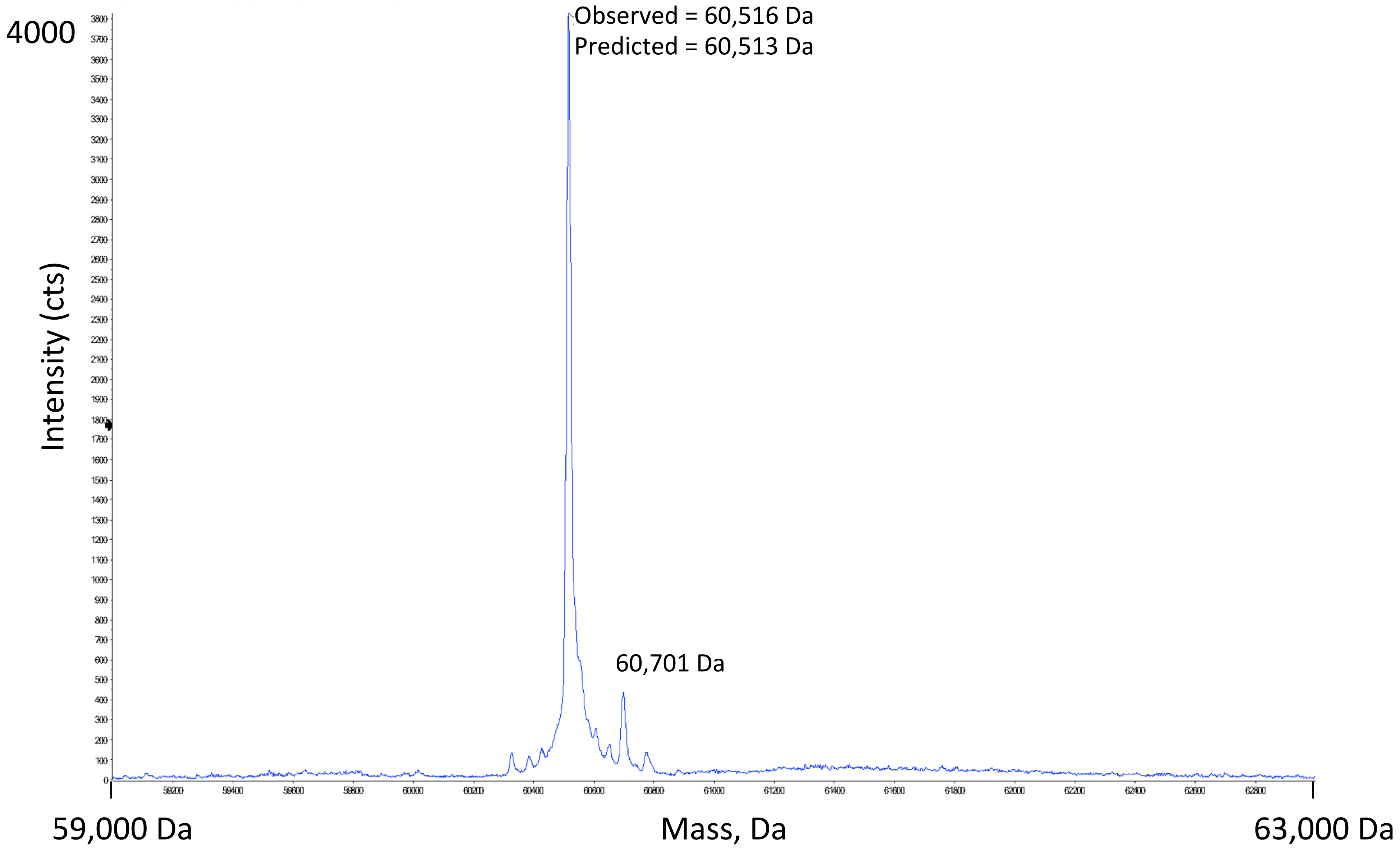

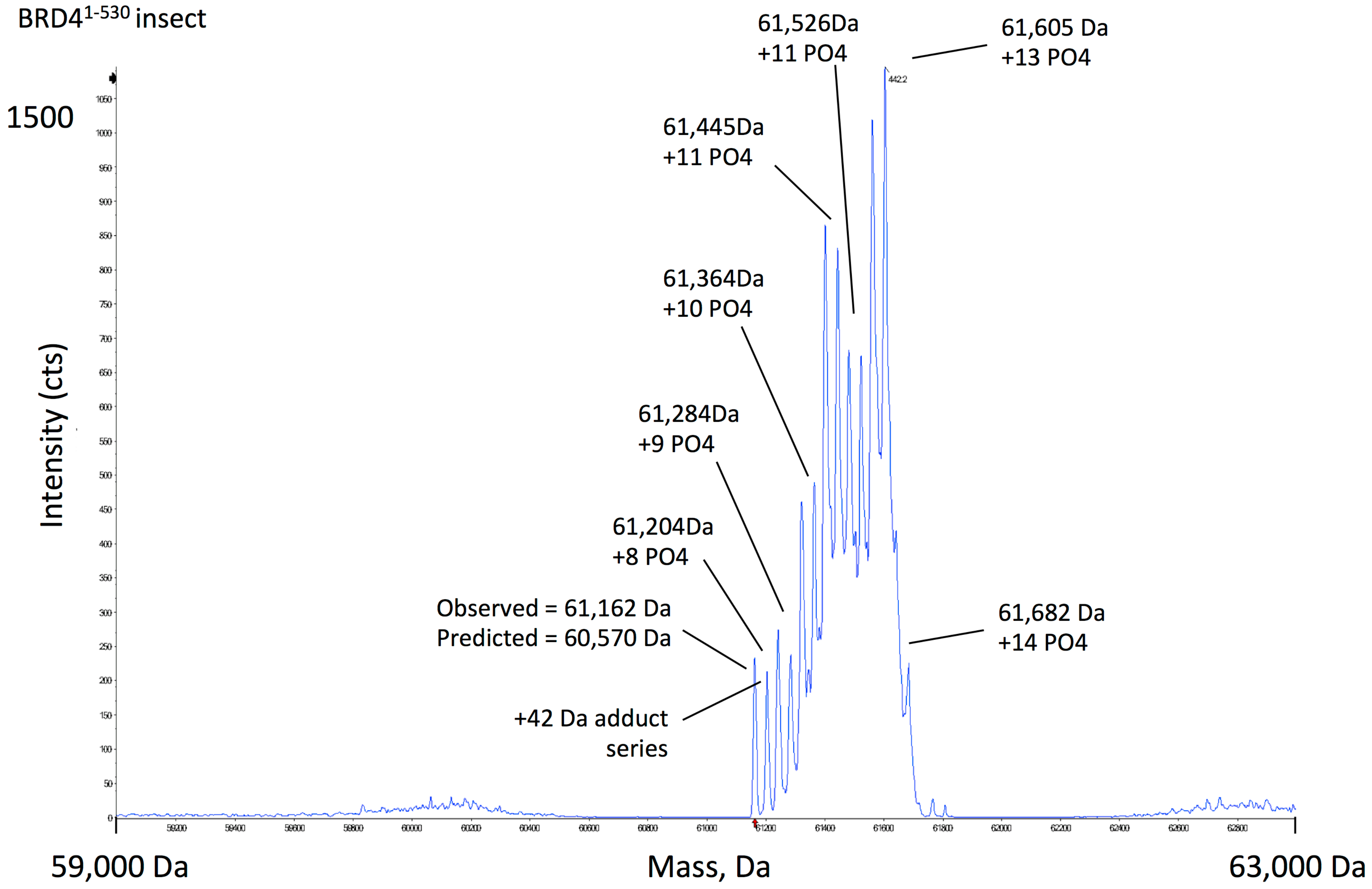

BRD4$^{1-530}$ insect
1500
Intensity (cts)
61,526Da
+11 PO4
61,605 Da
+13 PO4
61,445Da
+11 PO4
61,364Da
+10 PO4
61,284Da
+9 PO4
61,204Da
+8 PO4
Observed = 61,162 Da
Predicted = 60,570 Da
+42 Da adduct
series
61,682 Da
+14 PO4
59,000 Da
Mass, Da
63,000 Da

BRD4$^{1-530}$ CK2 phos.

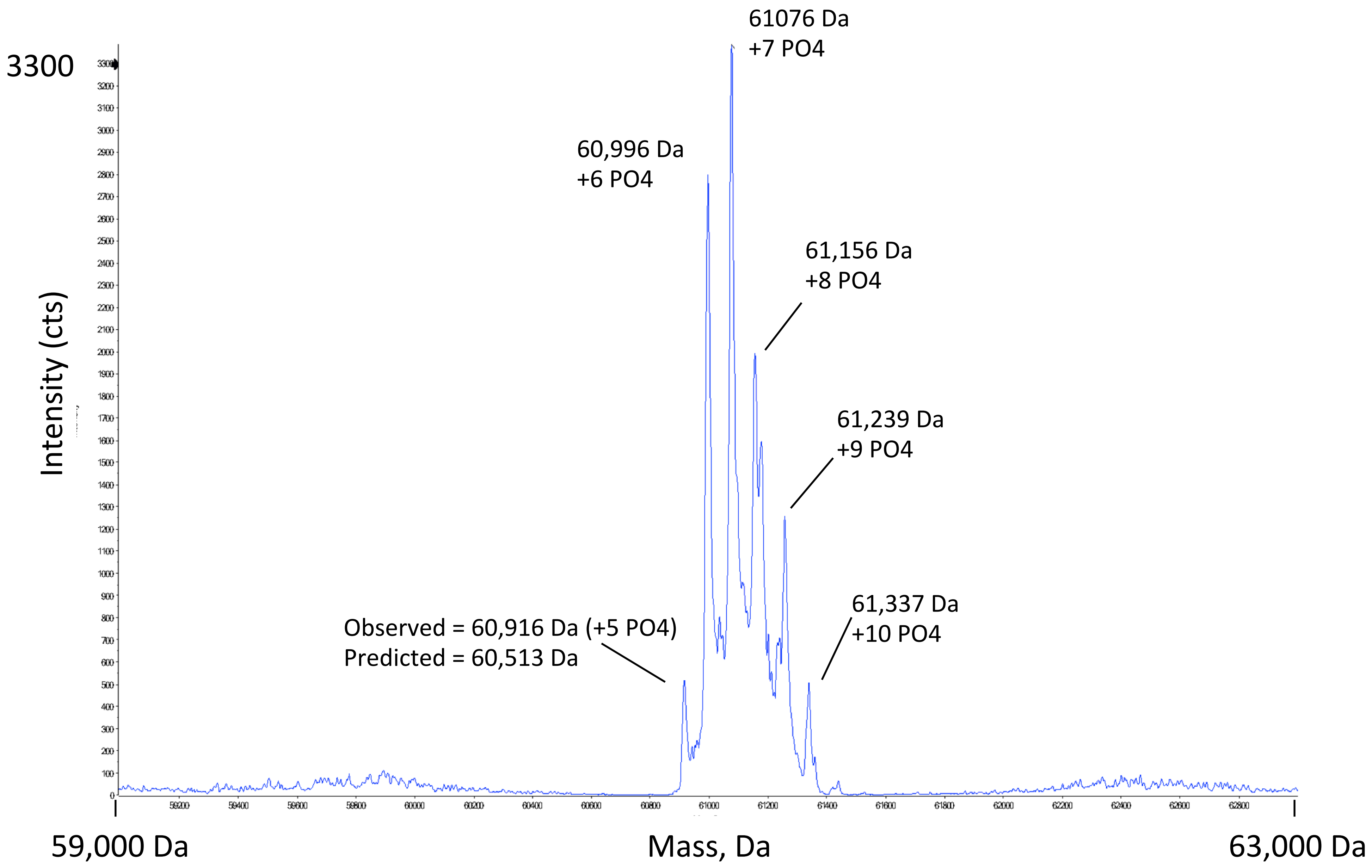

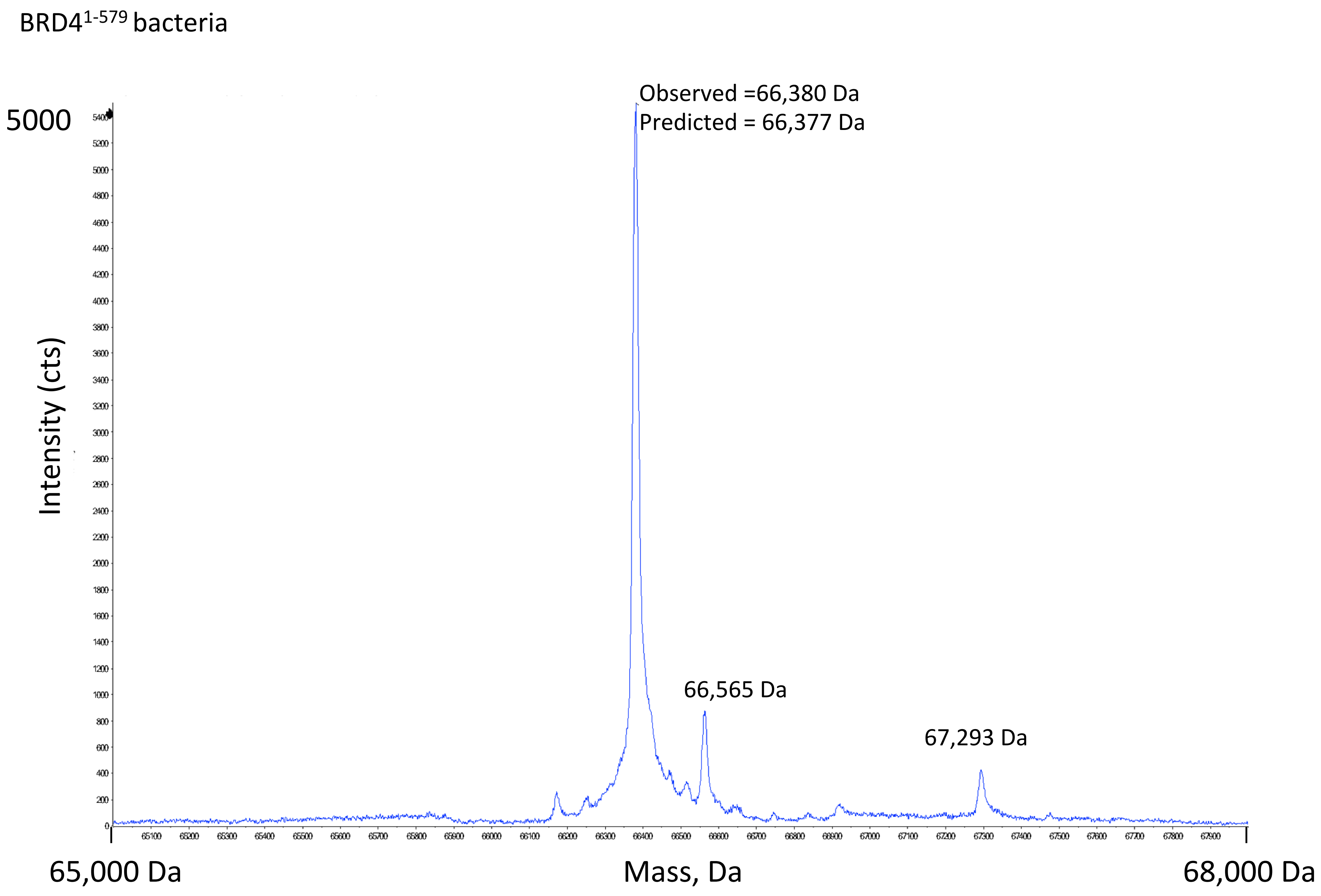
BRD4$^{1-579}$ bacteria
Observed =66,380 Da
Predicted = 66,377 Da
66,565 Da
67,293 Da
5000
Intensity (cts)
65,000 Da
Mass, Da
68,000 Da

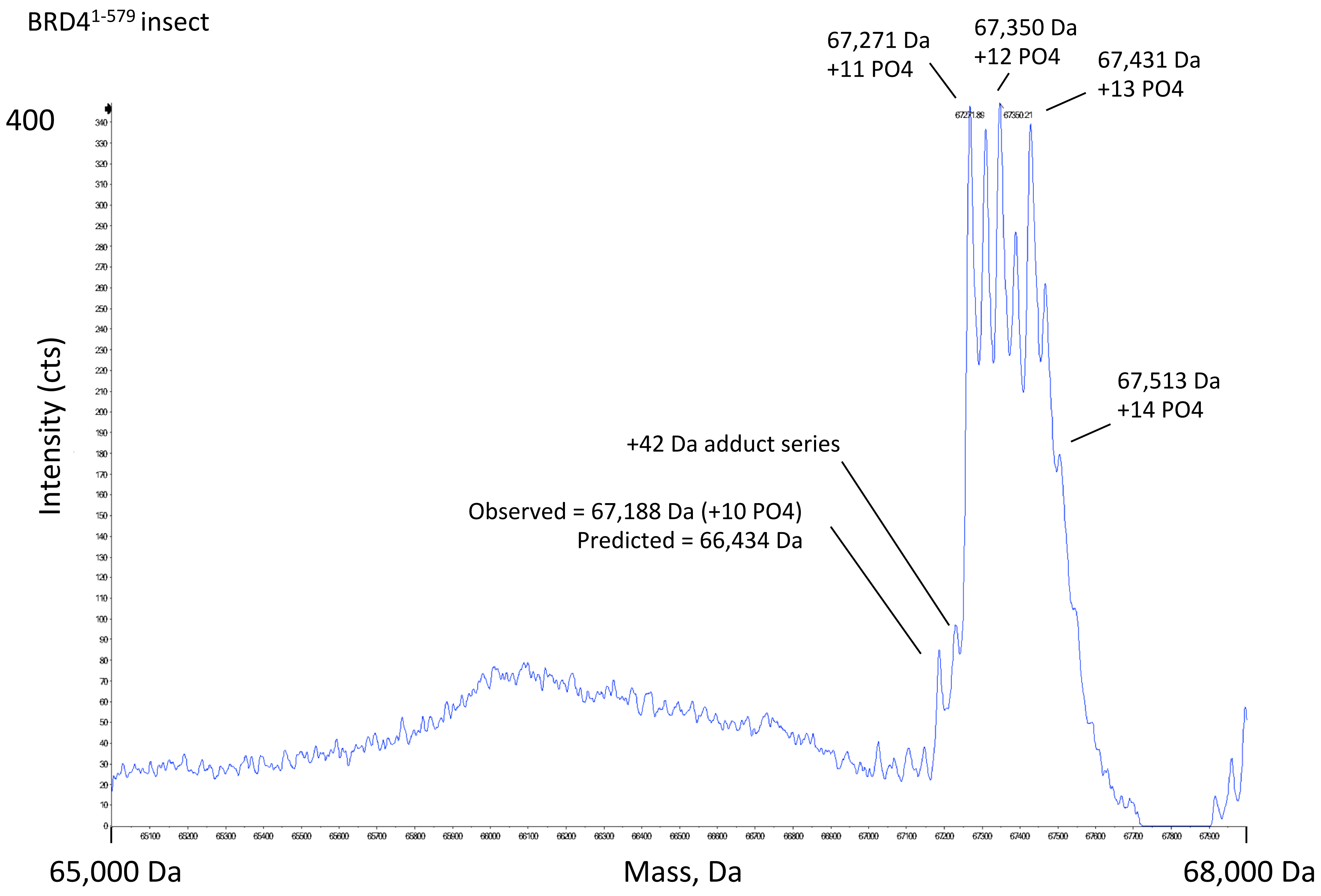

BRD4$^{1-579}$ insect
400
Intensity (cts)
67,271 Da
+11 PO4
67,350 Da
+12 PO4
67,431 Da
+13 PO4
67,513 Da
+14 PO4
+42 Da adduct series
Observed = 67,188 Da (+10 PO4)
Predicted = 66,434 Da
65,000 Da
Mass, Da
68,000 Da

BRD4$^{1-579}$ CK2 phos.

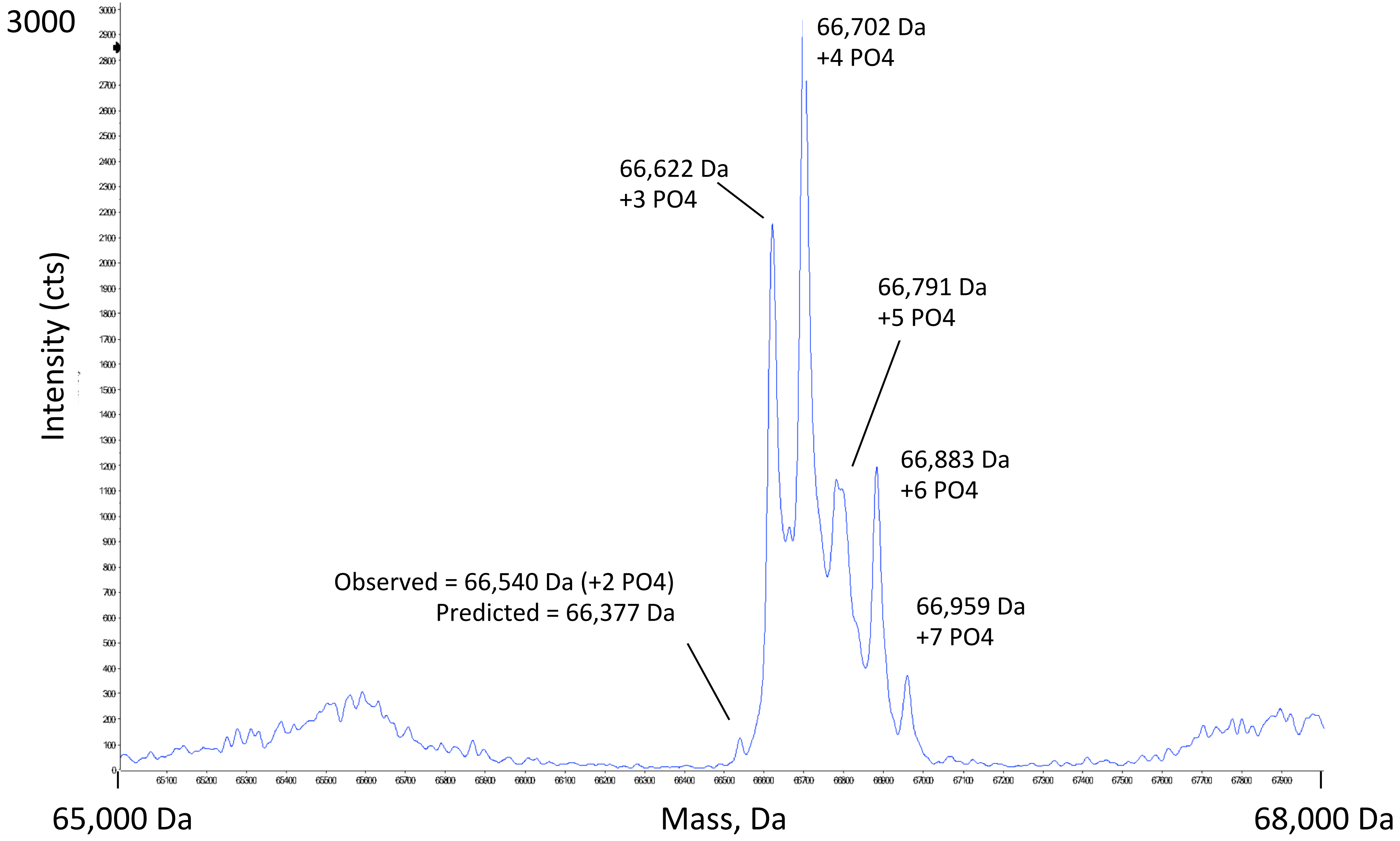

BRD4$^{1-722}$ bacteria

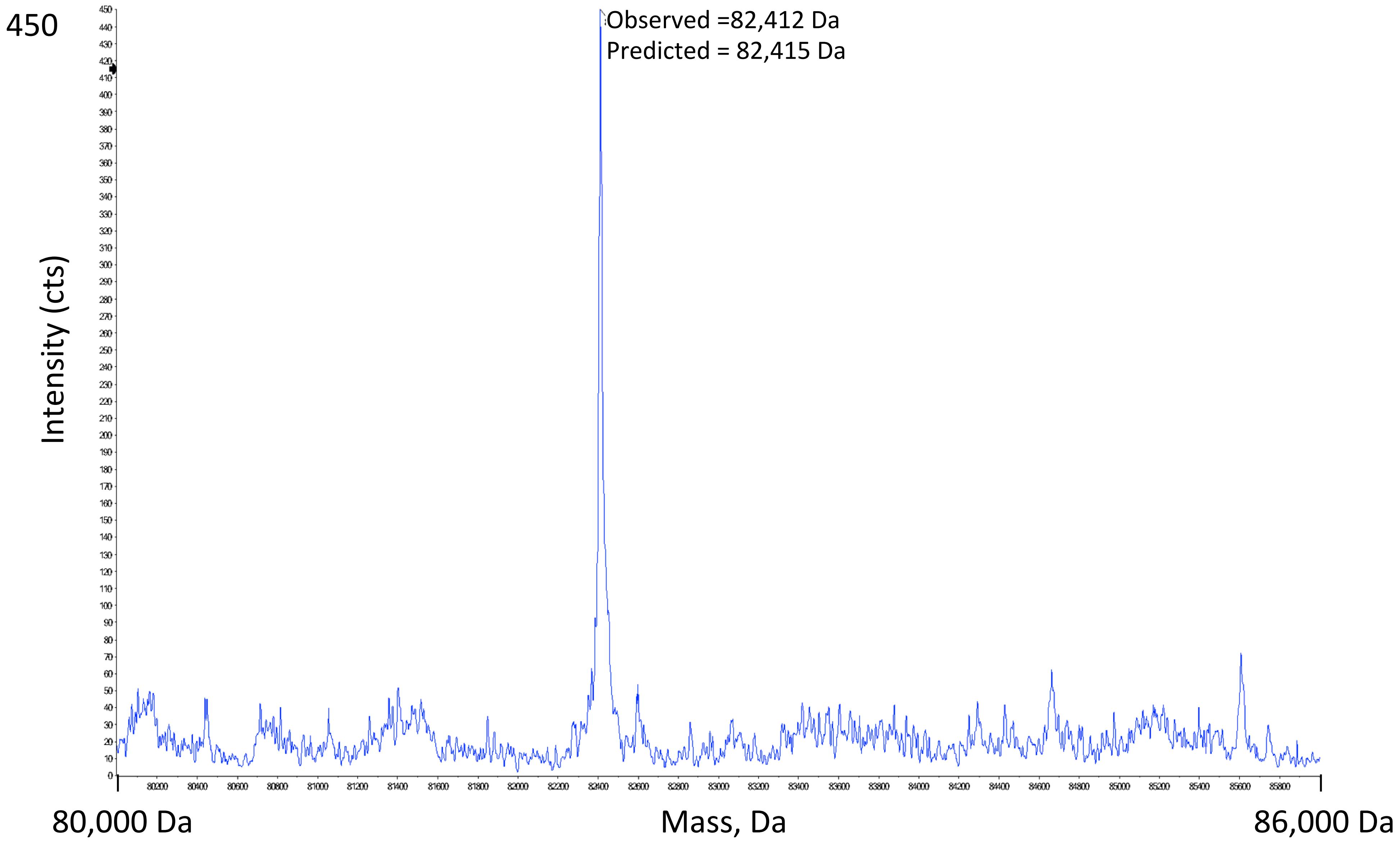

BRD4$^{1\text{-}722}$ insect

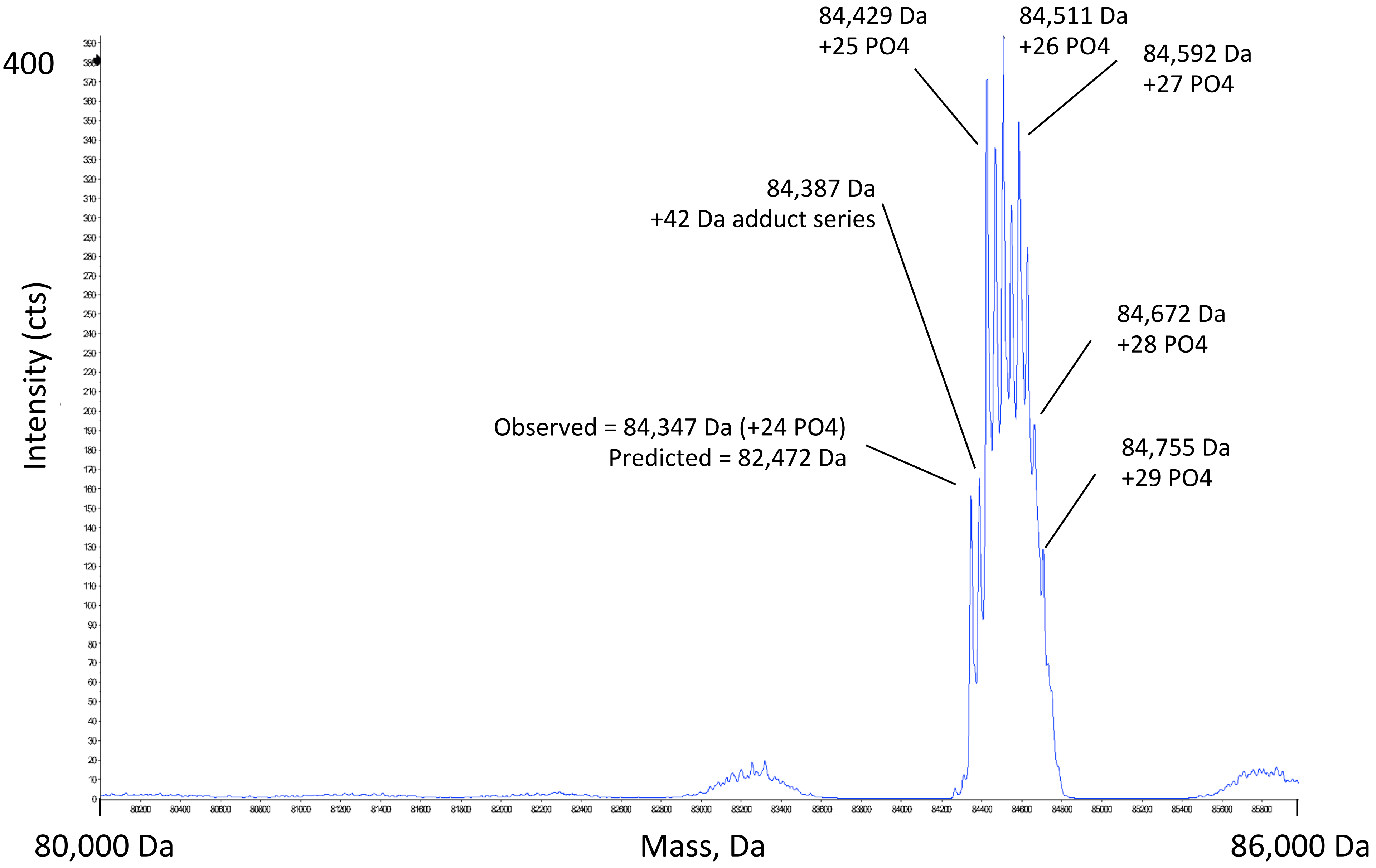

BRD4$^{1\text{-}722}$ CK2 phos.

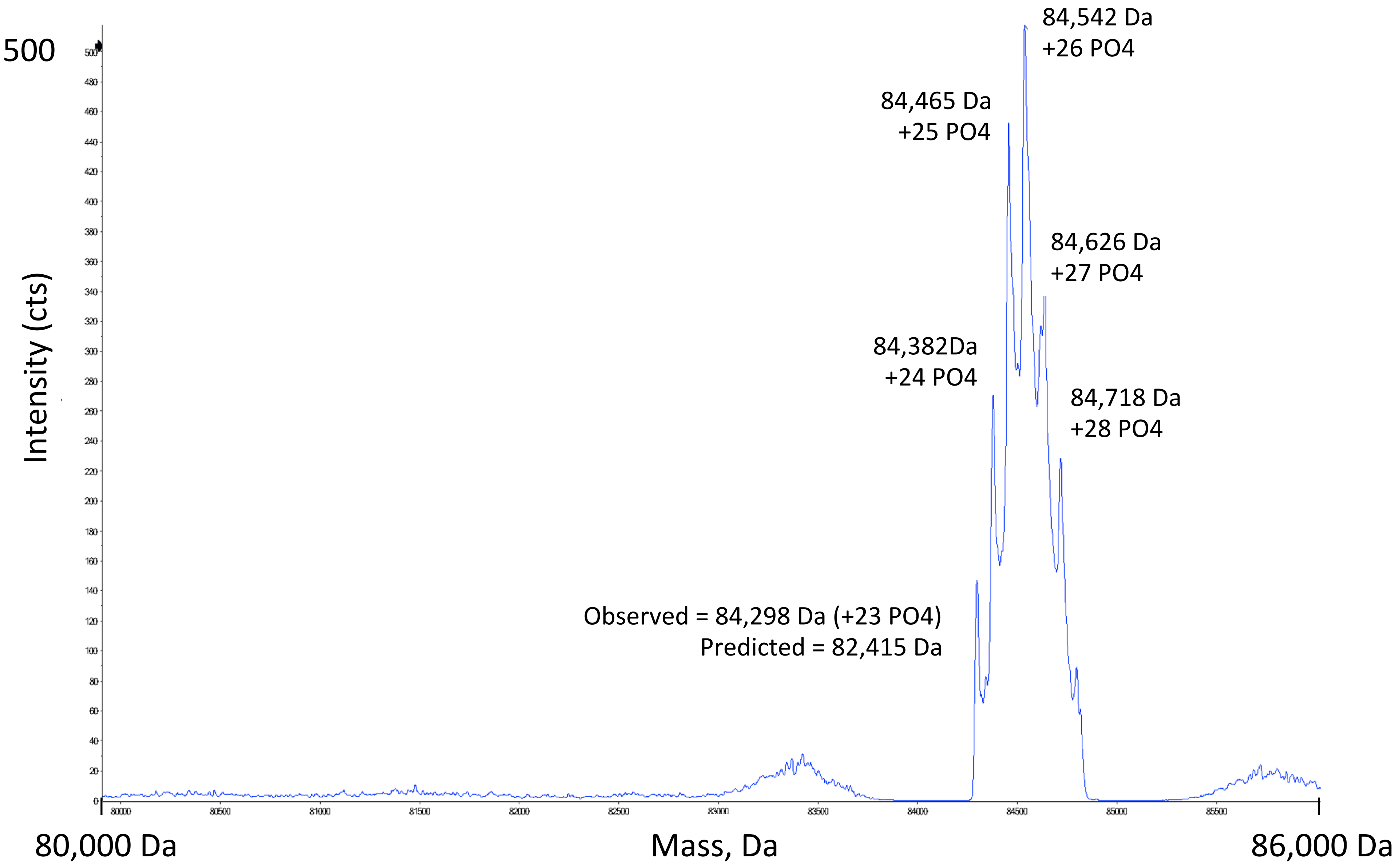

BRD4$^{1-722}$ 7A, CK2 phos.

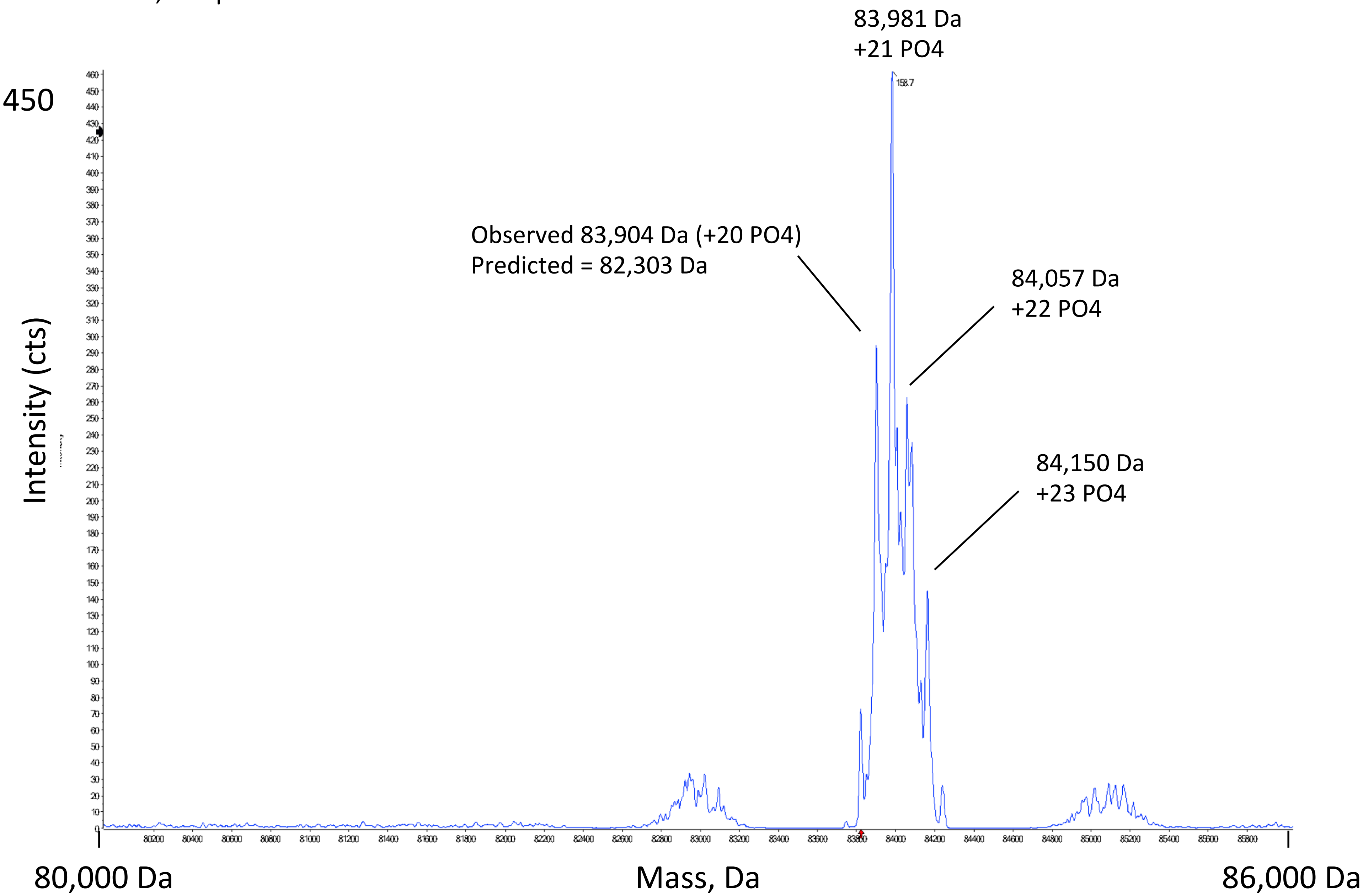

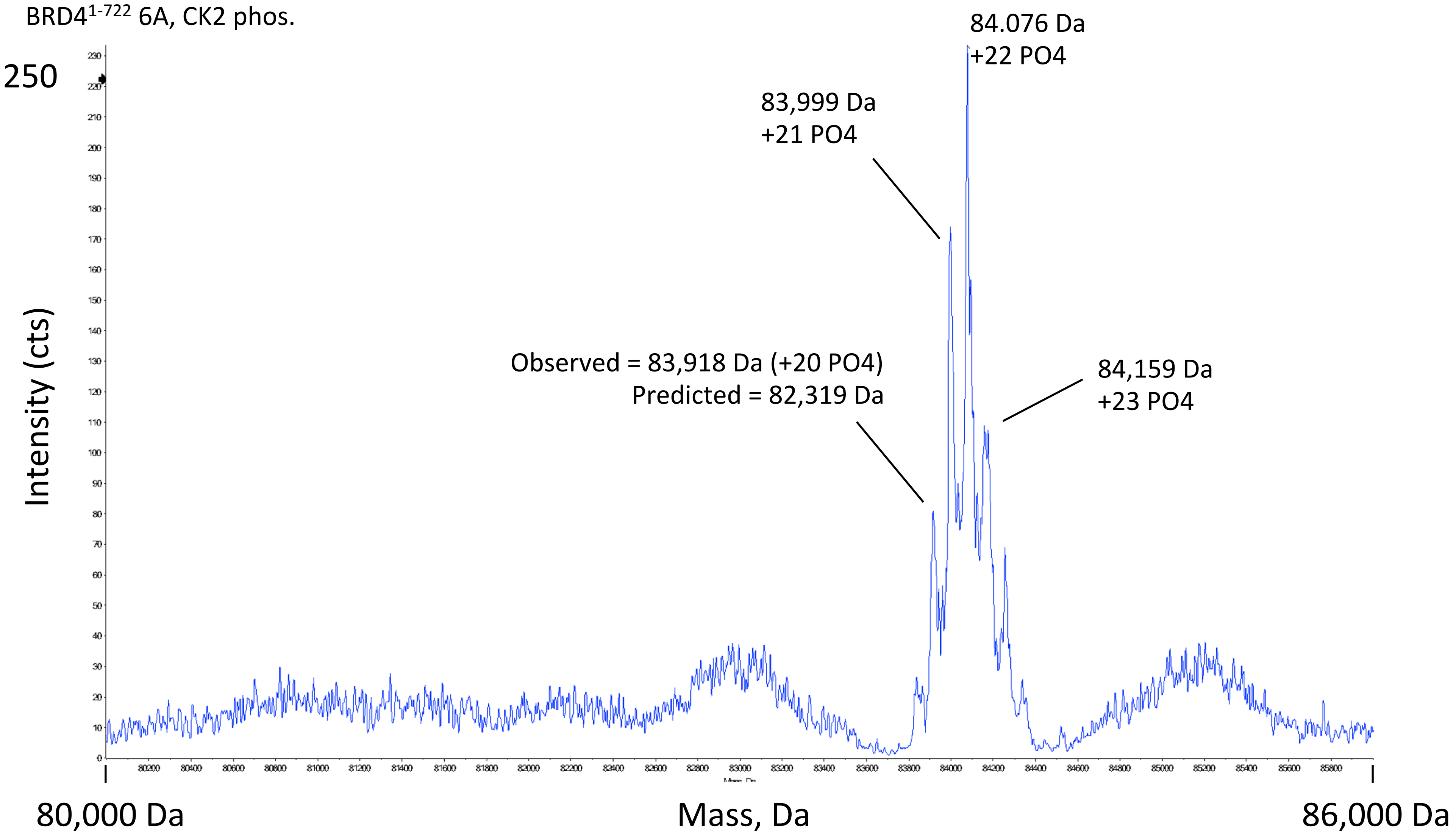

Malvezzi et al., Figure S1

Supplementary Figure 2

Primary sequence of BRD4 constructs. The amino acid sequences of bacterially expressed BRD4$^{1-530}$, BRD4$^{1-579}$, BRD4$^{1-722}$ are reported, including the N-terminal 6xHis-tag (blue) and TEV cleavage site (green). The amino acid sequences of the constructs produced in insect cells only differ in the N-terminal residues: MGHHHHHH instead of MHHHHHH. The sites predicted to be phosphorylated by CK2 (S/TxxE/D, where x is any residue) are highlighted in red.

BRD4$^{1-530}$

MHHHHHHGGGENLYFQGSAESGPGTRLRNLPVMGDGLETSQMSTTQAQAQPQPANAASTNPPPPETSNPNKPKRQTNQLQYLLRVVLKTL
WKHQFAWPFQQPVDAVKLNLPDYYKIIKTPMDMGTIKKRLENNYYWNAQECIQDFNTMFTNCYIYNKPGDDIVLMAEALEKLFLQKINELPTEE
TEIMIVQAKGRGRGRKETGTAKPGVSTVPNTTQASTPPQTQTPQPNPPPVQATPHPFPAVTPDLIVQTPVMTVVPPQPLQTPPPVPPQPQPP
PAPAPQPVQSHPPIIAATPQPVKTKKGVKRKADTTTPTTIDPIHEPPSLPPEPKTTKLGQRRESSRPVKPPKKDVPDSQQHPAPEKSSKVSEQL
KCCSGILKEMFAKKHAAYAWPFYKPVDVEALGLHDYCDIIKHPMDMSTIKSKLEAREYRDAQEFGADVRLMFSNCYKYNPPDHEVVAMARKL
QDVFEMRFAKMPDEPEEPVVAVSSPAVPPPTKVVAPPSSSDSSSDSSSDSDSSTDDSEEERAQRLAELQEQLKAVHEQLAALSQ

BRD4$^{1-579}$

MHHHHHHGGGENLYFQGSAESGPGTRLRNLPVMGDGLETSQMSTTQAQAQPQPANAASTNPPPPETSNPNKPKRQTNQLQYLLRVVLKTL
WKHQFAWPFQQPVDAVKLNLPDYYKIIKTPMDMGTIKKRLENNYYWNAQECIQDFNTMFTNCYIYNKPGDDIVLMAEALEKLFLQKINELPTEE
TEIMIVQAKGRGRGRKETGTAKPGVSTVPNTTQASTPPQTQTPQPNPPPVQATPHPFPAVTPDLIVQTPVMTVVPPQPLQTPPPVPPQPQPP
PAPAPQPVQSHPPIIAATPQPVKTKKGVKRKADTTTPTTIDPIHEPPSLPPEPKTTKLGQRRESSRPVKPPKKDVPDSQQHPAPEKSSKVSEQL
KCCSGILKEMFAKKHAAYAWPFYKPVDVEALGLHDYCDIIKHPMDMSTIKSKLEAREYRDAQEFGADVRLMFSNCYKYNPPDHEVVAMARKL
QDVFEMRFAKMPDEPEEPVVAVSSPAVPPPTKVVAPPSSSDSSSDSSSDSDSSTDDSEEERAQRLAELQEQLKAVHEQLAALSQPQQNKPK
KKEKDKKEKKKEKHKRKEEVEENKKSKAKEPPPKKTKKNNSS

BRD4$^{1-722}$

MHHHHHHGGGENLYFQGSAESGPGTRLRNLPVMGDGLETSQMSTTQAQAQPQPANAASTNPPPPETSNPNKPKRQTNQLQYLLRVVLKTL
WKHQFAWPFQQPVDAVKLNLPDYYKIIKTPMDMGTIKKRLENNYYWNAQECIQDFNTMFTNCYIYNKPGDDIVLMAEALEKLFLQKINELPTEE
TEIMIVQAKGRGRGRKETGTAKPGVSTVPNTTQASTPPQTQTPQPNPPPVQATPHPFPAVTPDLIVQTPVMTVVPPQPLQTPPPVPPQPQPP
PAPAPQPVQSHPPIIAATPQPVKTKKGVKRKADTTTPTTIDPIHEPPSLPPEPKTTKLGQRRESSRPVKPPKKDVPDSQQHPAPEKSSKVSEQL
KCCSGILKEMFAKKHAAYAWPFYKPVDVEALGLHDYCDIIKHPMDMSTIKSKLEAREYRDAQEFGADVRLMFSNCYKYNPPDHEVVAMARKL
QDVFEMRFAKMPDEPEEPVVAVSSPAVPPPTKVVAPPSSSDSSSDSSSDSDSSTDDSEEERAQRLAELQEQLKAVHEQLAALSQPQQNKPK
KKEKDKKEKKKEKHKRKEEVEENKKSKAKEPPPKKTKKNNSSNSNVSKKEPAPMKSKPPPTYESEEEDKCKPMSYEEKRQLSLDINKLPGEK
LGRVVHIIQSREPSLKNSNPDEIEIDFETLKPSTLRELERYVTSCLRKKRKPQAEKVDVIAGSSKMKGFSSSESESSSESSSSDSEDSETEMA

Supplementary Figure 3

BRD4$^{1-722}$ does not auto-phosphorylate in ADPglo assays. BRD4$^{1-722}$ purified from bacteria was tested for phosphorylation by CK2 or for auto-phosphorylation using the luminescent ADP detection assay ADPglo (Promega). An increasing signal is observed at increasing concentrations of BRD4$^{1-722}$ only in presence of the catalytic domain of CK2 subunit α, indicating that BRD4$^{1-722}$ can be phosphorylated by the kinase while it cannot auto-phosphorylate. The mean of n=3 with SE (standard error) is reported.

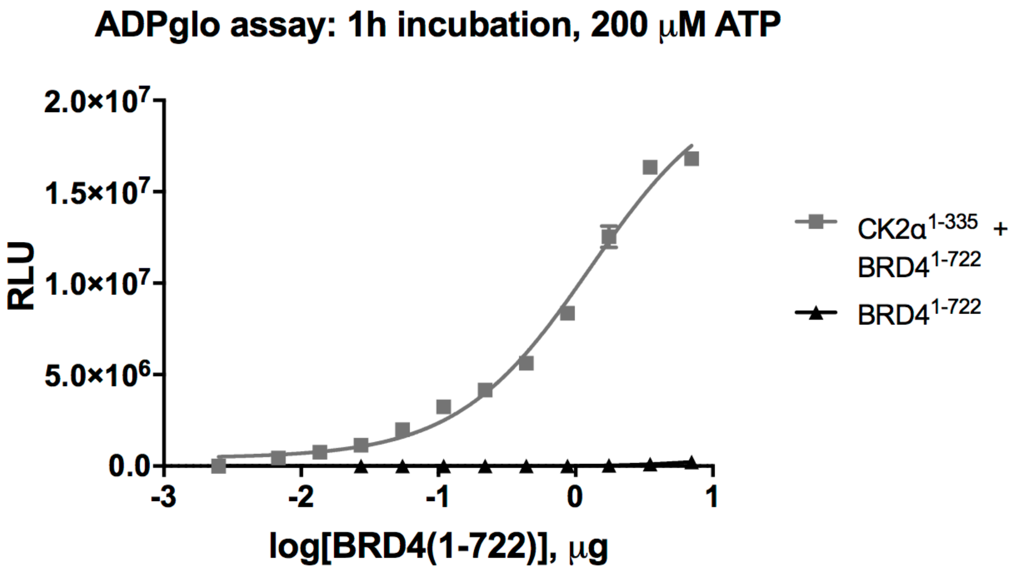

Supplementary Figure 4

Details of HDX-MS experiments. A. Sequence coverage of HDX-MS experiments for each of the constructs analysed. Only peptides identified in both phosphorylated and unphosphorylated samples were retained. **B.** Difference of deuterium uptake at each time point for every identified peptide between the sample purified from bacteria and the sample subjected to CK2 phosphorylation. A positive differential uptake indicates a higher deuterium uptake in the unphosphorylated sample. The threshold of 0.5 Da for significant difference is highlighted with a dotted line. In the final analysis, only peptides with changes above 0.5 Da and greater than 2.3x SD (standard deviation) were taken into account. The regions showing EX1 kinetics are highlighted with a dotted circle and they were not considered for the final analysis. For details on the calculation, see the Materials and Methods section.

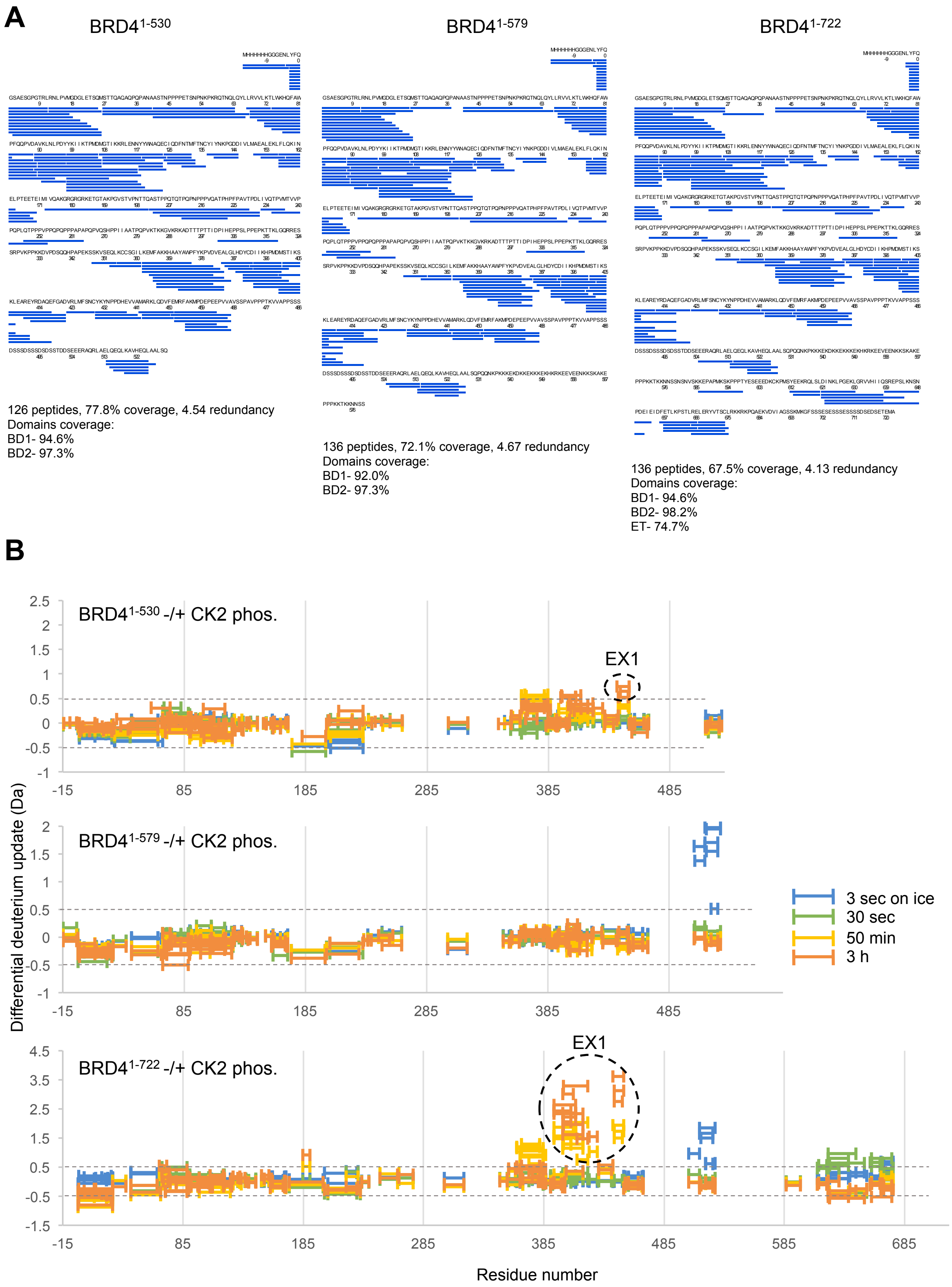
A
BRD4 1-530
BRD4 1-579
BRD4 1-722
126 peptides, 77.8% coverage, 4.54 redundancy
Domains coverage:
BD1- 94.6%
BD2- 97.3%
136 peptides, 72.1% coverage, 4.67 redundancy
Domains coverage:
BD1- 92.0%
BD2- 97.3%
136 peptides, 67.5% coverage, 4.13 redundancy
Domains coverage:
BD1- 94.6%
BD2- 98.2%
ET- 74.7%
B
BRD4 1-530 -/+ CK2 phos.
EX1
BRD4 1-579 -/+ CK2 phos.
BRD4 1-722 -/+ CK2 phos.
EX1
Differential deuterium update (Da)
Residue number
3 sec on ice
30 sec
50 min
3 h
-15
85
185
285
385
485
585
685

Supplementary Figure 5

Prediction of BRD4 oligomeric state. Output of the LOGICOIL algorithm for predicting the oligomeric state of coiled-coil sequences. The amino acid sequence of BRD4 isoform C (upper panel) or isoform A (lower panel) were used as input.

BRD4$^{1-722}$ (isoform C)

MARCOIL predicted region:

Sequence: SSDSDSSTDDSEEERAQRLAELQEQLKAVHEQLAALSQPQQNKPKKKEKDKKEKKKEKHKRKEEVEENKKSKAKEP

Register: gabcdefgabcdefgabcdefgabcdefgabcdefgabcdefgabcdefgabcdefgabcdefgabcdefgabcdb

Result of prediction:

Most probable state is ANTIPAPARALLEL DIMER

Second most probable state TETRAMER

	ANTI	PARA	TRIM	TETRA
Raw score is	1.04	0.94	0.85	0.99

MARCOIL output at 1 % threshold

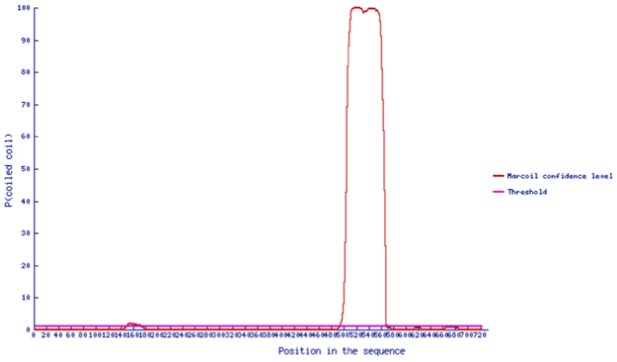

BRD4$^{1-1362}$ (isoform A)

MARCOIL predicted region: 1

Sequence: MAEALEKLFLQKINELPTEETEIM

Register: gabcdefgabcdefgabcdefgab

Result of prediction:

Most probable state is PARALLEL DIMER

Second most probable state ANTIPAPARALLEL DIMER

	ANTI	PARA	TRIM	TETRA
Raw score is	1	1.1	0.74	0.9

MARCOIL predicted region: 2

Sequence: SSDSDSSTDDSEEERAQRLAELQEQLKAVHEQLAALSQPQQNKPKKKEKDKKEKKKEKHKRKEEVEENKKSKAKEP

Register: gabcdefgabcdefgabcdefgabcdefgabcdefgabcdefgabcdefgabcdefgabcdefgabcdefgabcdb

Result of prediction:

Most probable state is ANTIPAPARALLEL DIMER

Second most probable state TETRAMER

	ANTI	PARA	TRIM	TETRA
Raw score is	1.04	0.94	0.85	0.99

MARCOIL output at 1 % threshold

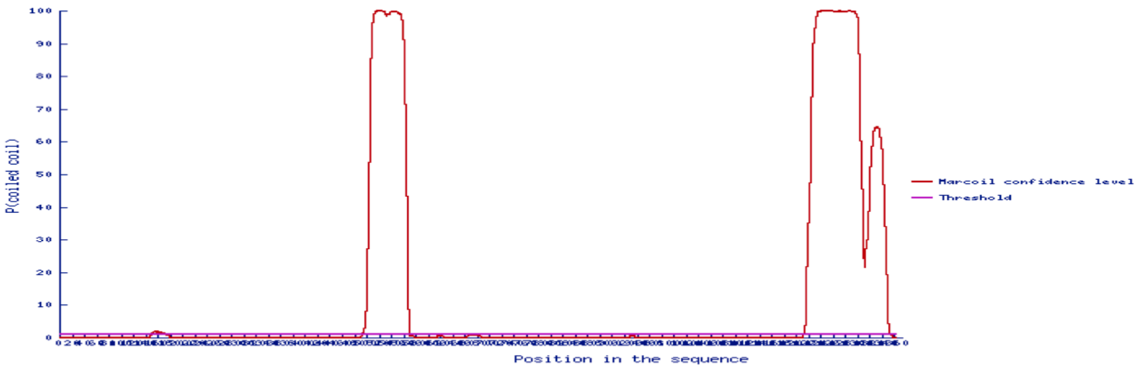

Malvezzi et al., Figure S5

Supplementary Figure 6

Minor changes in HDX in the domains of BRD4$^{1-722}$ upon phosphorylation by CK2. The average difference of deuterium uptake for each residue of BRD4$^{1-722}$ between the unphopshorylated and phosphorylated samples were plotted on the available crystal structures of BD1 (PDB ID: 2OSS) and BD2 (PDB ID: 2OUO) and on the NMR structure of the ET domain (PDB ID: 2JNS) of BRD4. A positive differential uptake is colored in blue, indicating a higher deuterium uptake in the unphosphorylated sample. Regions with no peptide coverage are colored in black.

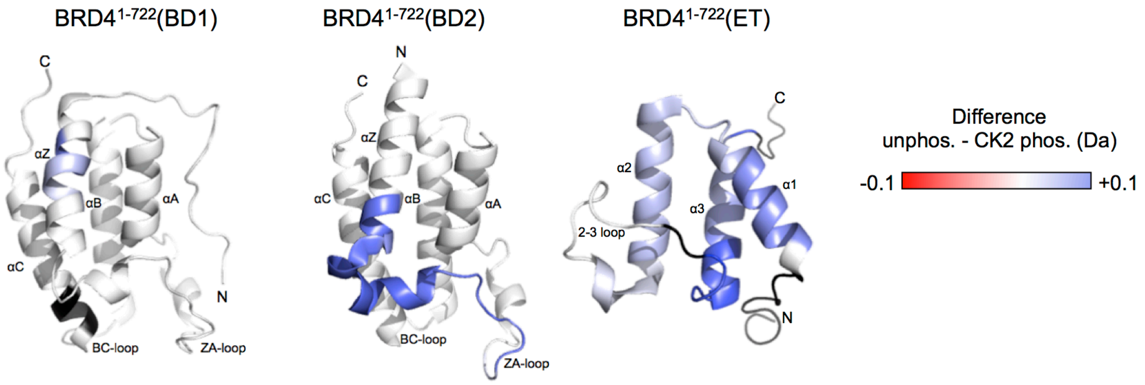

Supplementary Figure 7

EX1 kinetics in the BD2 domain of BRD4$^{1\text{-}722}$. A. Example of one of the 13 peptides in the BD2 domain of BRD4$^{1\text{-}722}$ showing EX1 kinetics at 50 min deuterium exchange time in the unphosphorylated protein. The m/z envelope of the 2+ ion of one experiment for each condition is analyzed using the software HX-Express2 (37) by fitting two Gaussian distributions. **B.** The relative deuterium uptake of peptide 400-409 can be plotted based on the overall centroid of the m/z envelope or on the calculated binomial Gaussian distributions. The presence of two distinct species having different deuterium uptake is observed only in the unphosphorylated sample. **C.** The regions showing a different deuterium uptake in the BD2 domain of BRD4$^{1\text{-}722}$ are highlighted in the crystal structure of the BD2 domain of BRD4 (PDB ID: 2OUO), colored based on the different type of kinetics of deuterium uptake. A list of the peptides clearly showing EX1 kinetics is reported on the right.

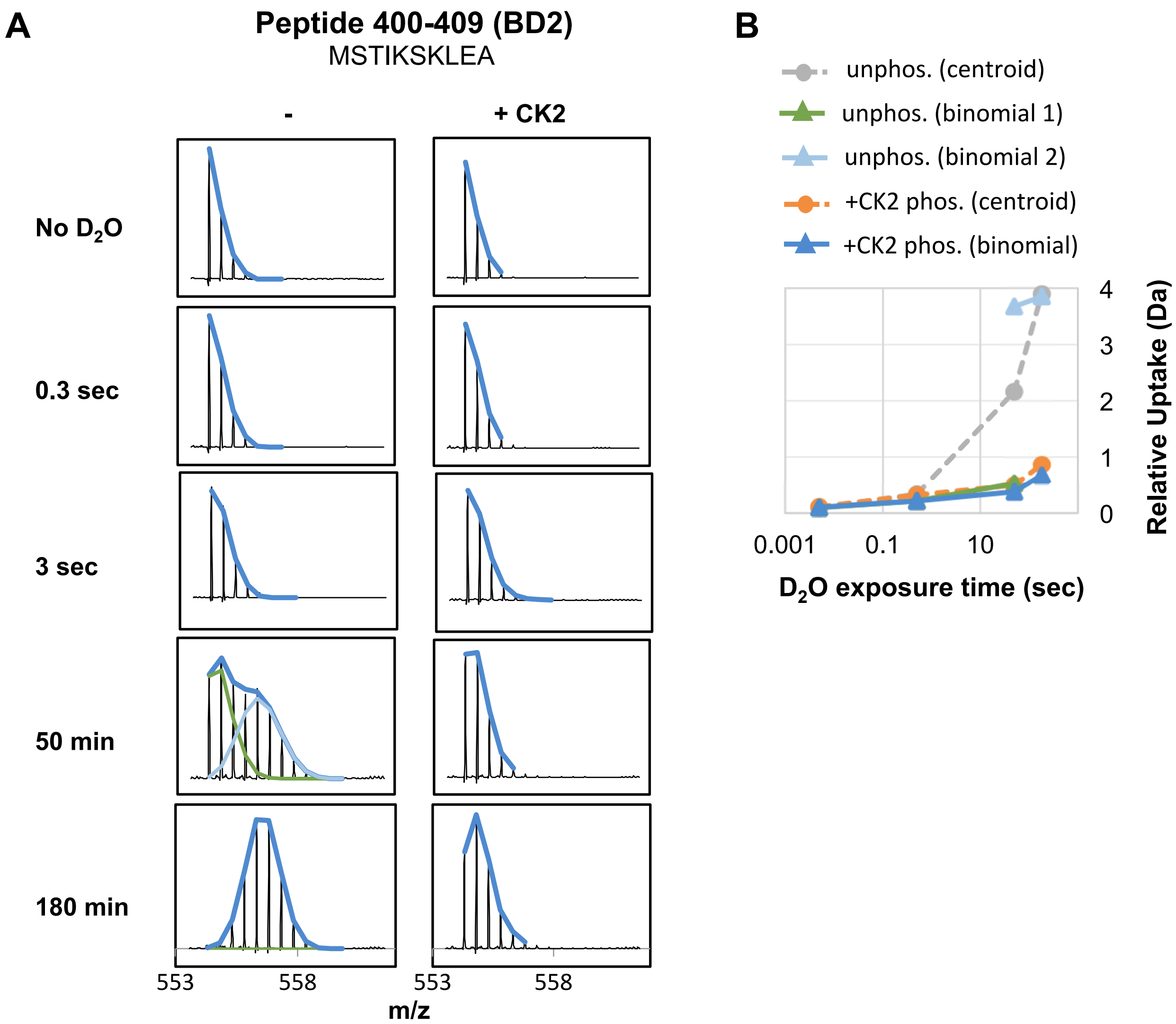

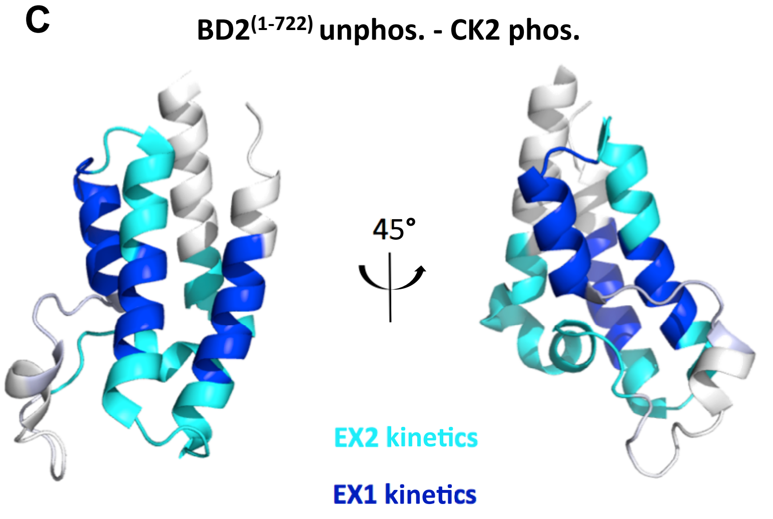

Peptides with EX1 kinetics (aa)
393-407
393-417
393-411
400-411
400-407
400-409
401-411
401-417
401-421
422-429
442-451
443-450
443-451

Supplementary Figure 8

Preparation of BRD4$^{1\text{-}722}$ cross-linked samples for XL-MS. Coomassie-stained SDS-PAGE of 2.2 μM BRD4$^{1\text{-}722}$ unphosphorylated or CK2 phosphorylated after incubation with increasing amounts of cross-linker Bis(sulfosuccinimidyl) suberate BS3 (0, 21x, 62x, 185x, 555x, 1666x, 5000x). In the right panel, the intensity of the dimer band is reported normalized to the intensity of the total protein without BS3, analysed with ImageJ.

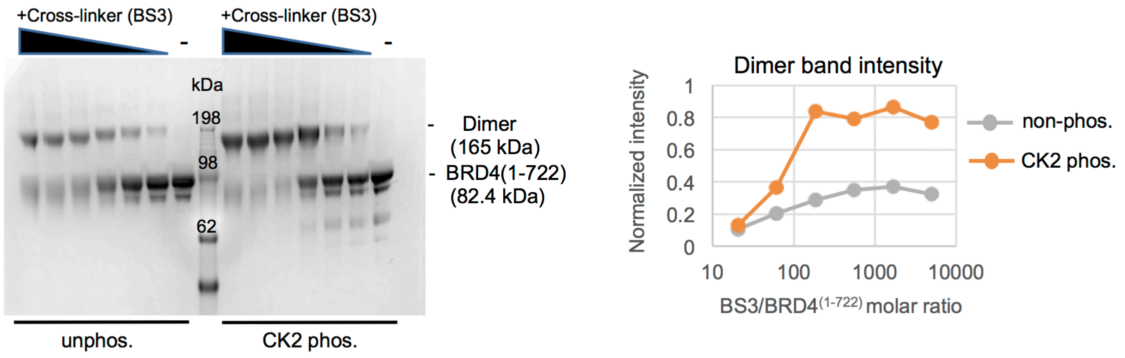

Supplementary Figure 9

Intact mass spectrometry of BRD4$^{1\text{-}722}$ (Δ506-530). The phosphorylation state of BRD4$^{1\text{-}722}$ (Δ506-530) purified from insect cells with and without treatment with λ-phosphatase was analysed by mass spectrometry. The number of phospho-groups calculated based on the difference between the observed and the predicted molecular weight is shown at each corresponding peak. In both samples, a parallel set of peaks is indicated, corresponding to the acetylated protein differentially phosphorylated (+42 Da difference).

BRD4[1-722](Δ506-530)
Detected mass= 82434.4 → expected mass (80404.87) + 25 phosphorylations (or 27 based on dephosphorylated sample mass).

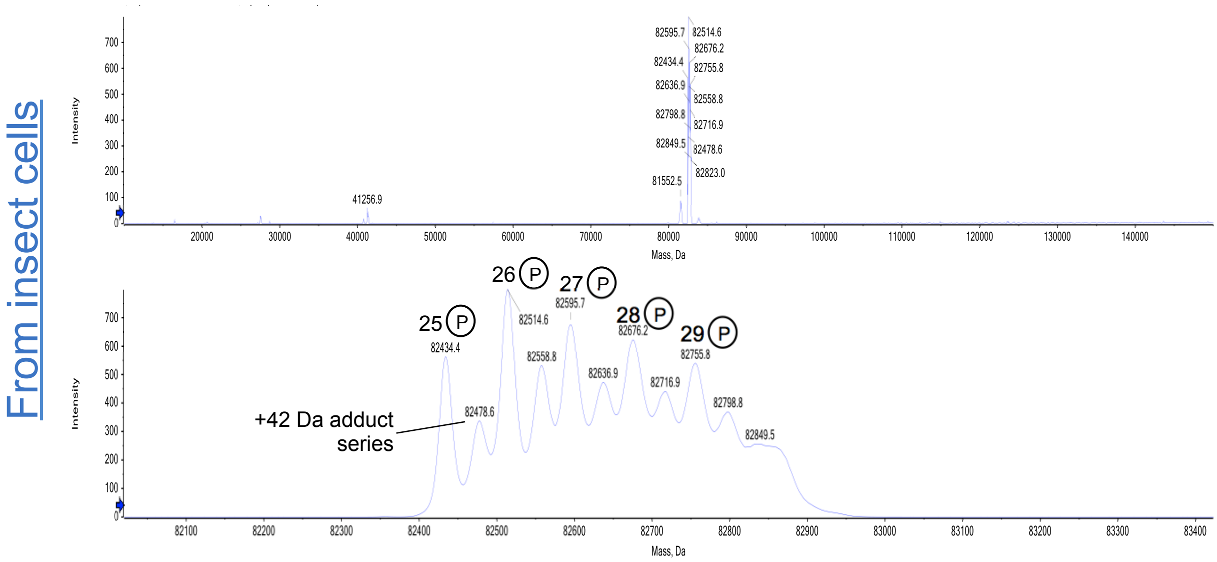

BRD4[1-722](Δ506-530)
Detected mass= 80234.7 Da → expected mass (80404.87) -170.17 Da

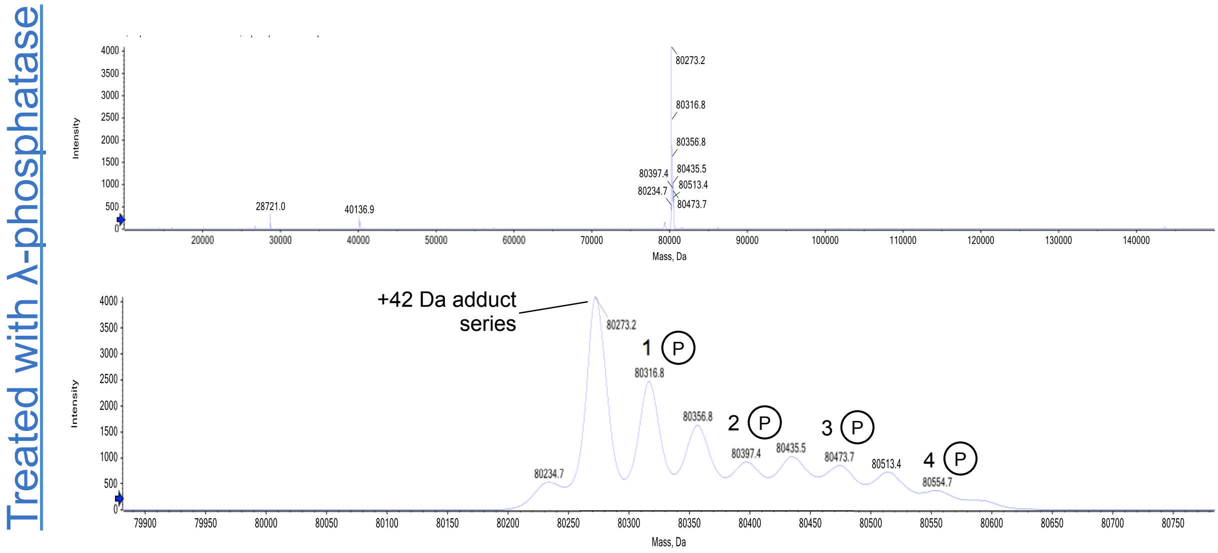

Malvezzi et al., Figure S9

Supplementary Figure 10

Monovalent (I-BET) and series of biBETs used in the study.

I-BET –
monovalent

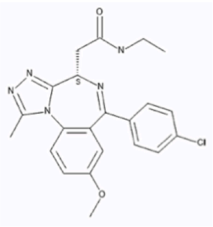

6

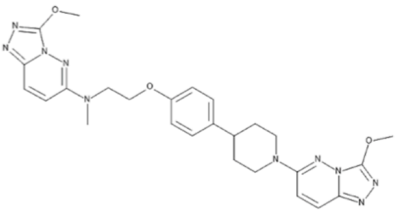

7

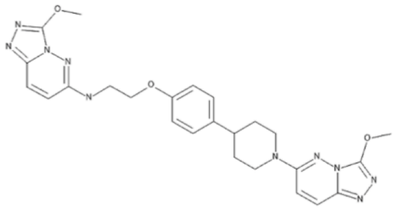

9

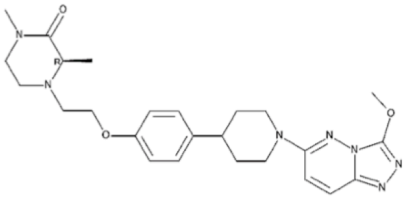

10

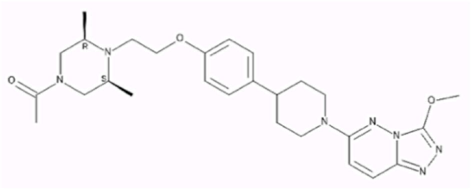

Uncropped gels

Uncropped gel corresponding to Figure 1B.

Figure 1B

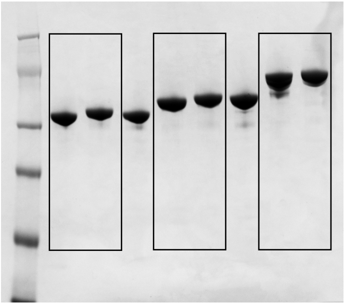

Supplement: Supplementary file 1 — Supplementary Information [file 42003_2021_2750_MOESM1_ESM.pdf]
